# RNA-Sequencing Reveals Unique Transcriptional Signatures of Running and Running-Independent Environmental Enrichment in the Adult Mouse Dentate Gyrus

**DOI:** 10.3389/fnmol.2018.00126

**Published:** 2018-04-13

**Authors:** Catherine-Alexandra Grégoire, Stephanie Tobin, Brianna L. Goldenstein, Éric Samarut, Andréanne Leclerc, Anne Aumont, Pierre Drapeau, Stephanie Fulton, Karl J. L. Fernandes

**Affiliations:** ^1^Research Center of the University of Montreal Hospital, University of Montreal, Montreal, QC, Canada; ^2^CNS Research Group, University of Montreal, Montreal, QC, Canada; ^3^Department of Pathology and Cell Biology, Faculty of Medicine, University of Montreal, Montreal, QC, Canada; ^4^Department of Nutrition, Faculty of Medicine, University of Montreal, Montreal, QC, Canada; ^5^Department of Neurosciences, Faculty of Medicine, University of Montreal, Montreal, QC, Canada

**Keywords:** dentate gyrus, exercise, RNA-sequencing, social environment, hippocampal neurogenesis, environmental enrichment

## Abstract

Environmental enrichment (EE) is a powerful stimulus of brain plasticity and is among the most accessible treatment options for brain disease. In rodents, EE is modeled using multi-factorial environments that include running, social interactions, and/or complex surroundings. Here, we show that running and running-independent EE differentially affect the hippocampal dentate gyrus (DG), a brain region critical for learning and memory. Outbred male CD1 mice housed individually with a voluntary running disk showed improved spatial memory in the radial arm maze compared to individually- or socially-housed mice with a locked disk. We therefore used RNA sequencing to perform an unbiased interrogation of DG gene expression in mice exposed to either a voluntary running disk (RUN), a locked disk (LD), or a locked disk plus social enrichment and tunnels [i.e., a running-independent complex environment (CE)]. RNA sequencing revealed that RUN and CE mice showed distinct, non-overlapping patterns of transcriptomic changes versus the LD control. Bio-informatics uncovered that the RUN and CE environments modulate separate transcriptional networks, biological processes, cellular compartments and molecular pathways, with RUN preferentially regulating synaptic and growth-related pathways and CE altering extracellular matrix-related functions. Within the RUN group, high-distance runners also showed selective stress pathway alterations that correlated with a drastic decline in overall transcriptional changes, suggesting that excess running causes a stress-induced suppression of running’s genetic effects. Our findings reveal stimulus-dependent transcriptional signatures of EE on the DG, and provide a resource for generating unbiased, data-driven hypotheses for novel mediators of EE-induced cognitive changes.

## Introduction

The hippocampus is a component of the brain’s limbic system that has been implicated in higher cognitive processes such as learning and memory, spatial navigation, and emotional regulation (Frankland et al., 1998; Bannerman et al., 2003; Kheirbek et al., 2013; Trivino-Paredes et al., 2016). Its cellular organization includes a prominent tri-synaptic circuit that spans across its two main structures, the dentate gyrus (DG) and Ammon’s horn (CA), with the neocortex serving as the principal source of inputs and outputs (Anderson et al., 2007). The precise mechanisms by which the hippocampal tri-synaptic circuit mediates higher cognitive processes remains incompletely understood, but it is likely that the DG plays a prominent role in hippocampal-dependent functions (Arruda-Carvalho et al., 2011; Kheirbek et al., 2013; Vukovic et al., 2013). Anatomically, synapses between axons of entorhinal cortex neurons and DG granule cells are the first synapses of the tri-synaptic circuit and represent the principal gateway to hippocampal function (Anderson et al., 2007). By virtue of its dense granule cell layer, which vastly outnumbers inputs from the cortex, the DG performs a pattern separation function that enables closely related inputs to be encoded distinctly within the CA3 layer (Deng et al., 2010; Aimone et al., 2014). The DG is also the sole region of the hippocampus in which neural stem cells (NSCs) remain active throughout life (Gage, 2000; Kriegstein and Alvarez-Buylla, 2009; Bond et al., 2015). NSCs continuously produce new, highly plastic granule neurons through the process of adult neurogenesis, and ablation of these newly generated granule cells in rodents compromises normal learning and memory as well as regulation of stress and emotion (Kheirbek et al., 2013; Aimone, 2016).

Hippocampal and wider brain functions are powerfully enhanced by environmental enrichment (EE) (Hebb, 1947; Rosenzweig et al., 1962; La Torre, 1968; Bennett et al., 1969; Kempermann et al., 1997; Gelfo et al., 2017). EE typically involves exposure to diverse multisensory stimuli, including physical activity, social enrichment, and spatial complexity, which trigger the changes in growth factor synthesis, dendritic modifications, synaptic plasticity, and neurogenesis that are thought to underlie improvements in brain function (Nithianantharajah and Hannan, 2006). Physical activity is a particularly critical component of EE, with running having been directly established as the stimulus of EE-induced DG neurogenesis in rodents (Kobilo et al., 2011; Mustroph et al., 2012; Gregoire et al., 2014). Moreover, physical activity paradigms in humans can substantially improve cognition during aging, mild cognitive impairment, and dementia (Baker et al., 2010; Carvalho et al., 2014; Strohle et al., 2015; Groot et al., 2016; Bettio et al., 2017). The running-independent components of EE also measurably impact rodent brain function, increasing depolarization-associated c-fos expression within the DG while decreasing circulating levels of the stress hormone corticosterone (Gregoire et al., 2014), whose receptors are richly expressed in the brain. Thus, running and running-independent components of EE exert distinct and separable physiological effects on the DG.

The cellular and molecular mechanisms mediating EE-induced changes in DG function remain only partially understood. Previous candidate-based studies have identified growth factors such as brain-derived neurotrophic factor (BDNF), fibroblast growth factor (FGF)-2, insulin-like growth factor (IGF)-1, and vascular endothelial growth factor (VEGF) as molecular mediators of EE effects on the DG. To investigate this question in a more unbiased fashion, here we used an RNA sequencing (RNA-Seq) strategy to perform a genome-wide assessment of EE-induced transcriptional changes. Our data illuminate global patterns of biological functions, cellular compartments, and genetic pathways modified within the DG in response to running and running-independent EE paradigms.

## Materials and Methods

### Mice

Animal procedures were approved by the institutional animal care committees of the University of Montreal and the CRCHUM and were conducted in accordance with the guidelines of the Canadian Council of Animal Care. Experiments were performed using 3-month-old male CD1 mice (Charles River Laboratories, Saint-Constant, QC, Canada) that were housed in a reverse 12-h light/dark cycle (lights on at 10:00 pm and off at 10:00 am) with food and water *ad libitum*. For behavioral testing, a total of 44 mice were used (14–15/group), which were obtained using two separate cohorts of mice. For transcriptomic studies, a total of 24 mice were used (8/group).

### Environmental Enrichment

Since our previous work did not detect any additive effects of running and running-independent EE on DG neurogenesis, DG c-fos expression, or levels of circulating corticosterone (Gregoire et al., 2014), for the present study, we used three experimental groups in behavioral and transcriptomic experiments: an individually-housed voluntary running group (RUN group), an individually-housed locked disk control group (LD group), and a socially-housed running-independent EE group. In the behavioral experiments, the running-independent EE group was housed with a locked disk+social housing (SOC group), while in the transcriptomic experiments it was housed with a LD+social housing+tunnels, referred to here as the complex environment (CE group). These groups are detailed below.

RUN mice were individually housed in rat cages (39.3 cm × 28.5 cm × 19.4 cm) that contained a running disk (Red mouse igloo, K3327, and amber fastrac running disk, 7.5 cm in diameter, K3250, Bio-Serv, Frenchtown, NJ, United States). LD mice were housed identically to RUN mice except that the running disk was glued to prevent voluntary running. SOC mice were housed identically to LD mice except that they were in groups of three mice per cage. CE mice were housed identically to SOC mice except that the cages also contained tunnels whose locations were altered daily, providing a socially-enriched CE. All environments contained nesting material and basic litter (Beta chip, Nepco). Running cages were outfitted with odometers (Sigma BC509) to measure the running distance.

### Behavioral Testing

Mice used for behavioral studies were processed in two cohorts (*n* = 18 and *n* = 26) in order to obtain sufficient total numbers of animals. Each cohort contained mice from all three experimental groups. Basal locomotor activity was assessed by measuring the total distance traveled during the 10-min habituation phase of the novel object recognition (NOR) test.

#### Delayed Non-matching to Place Radial Arm Maze (RAM)

**FIGURE 1:**
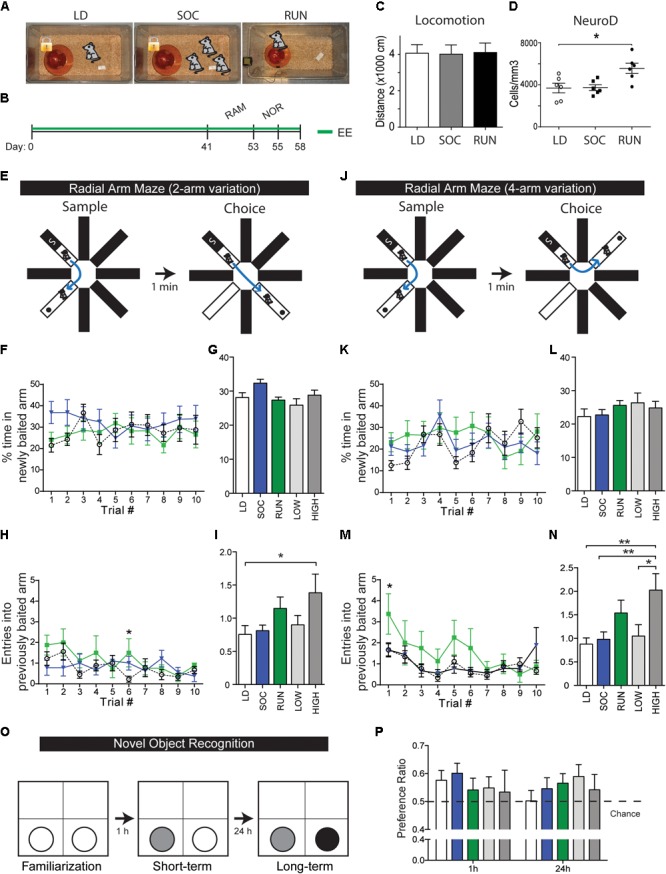
Behavioral testing of environmentally enriched mice. **(A)** EE groups. **(B)** Timeline of experiment. Mice spent 41 days housed in their respective enriched environments, then spent another additional 14 days undergoing behavioral testing in the radial arm maze (RAM, 12 days) and novel object recognition test (NOR, 2 days). Enriched environments were maintained during the testing period, and animals were sacrificed for tissue analysis 3 days following the testing period. **(C)** Quantification of open field locomotor activity. Note that there are no differences in baseline locomotion. **(D)** Quantifications of NeuroD+ neuroblasts within the DG at the end of the behavioral experiment. Note that NeuroD+ neuroblasts were unchanged between LD and SOC groups (*p* = 0.9633) and were significantly increased in the RUN group (*p* = 0.0117 versus LD). One-way ANOVA. **(E–N)** Radial arm maze. Illustrations of the 2- and 4-arm variations of the maze test **(E,J)**. *% time in newly baited arm*, shown plotted daily **(F,K)** or as an average over the 10-day period **(G,L)**, *n* = 14–15/group from two cohorts. *Entries into previously baited arm*, shown plotted daily **(H,M)** or as an average over the 10-day period **(I,N)**, *n* = 8–9/group from one cohort. Note that high-distance runners show a significant increase in mean entries in both arm variations (2-arm: LD vs. HIGH *p* = 0.0288, 4-arm: LD vs. HIGH *p* = 0.0013, SOC vs. HIGH *p* = 0.0046, LOW vs. HIGH *p* = 0.0452). Two-way ANOVA **(F,H,K,M)** and one-way ANOVA **(G,I,L,N)**. **(O,P)** Novel object recognition. Illustration of object changes during the familiarization, short-term memory, and long-term memory phases of the test **(O)**. Preference ratios, defined as proportion of time spent with the new object, at the short-term and long-term memory testing phases **(P)**, *n* = 14–15/group. ^∗^*P* ≤ 0.05; ^∗∗^*P* ≤ 0.01.

Mice received a small amount of palatable food (sugar pellets, BioServ) in their home cages several days before training to become familiar with the reward. Disks and food were removed 2 h before testing (8:00 am) and were returned following testing. The RAM consisted of eight (37 cm long) equidistantly spaced arms radiating from a small octagonal platform linked to a detection system (Med Associates). The delayed non-matching to place test is designed to assess the ability of a mouse to discriminate an originally baited sample arm (familiar-first sugar bated arm) from a newly baited choice arm (Clelland et al., 2009). For training and testing, animals were only able to access the center and one or two radial arms. For training, mice were given two pre-exposures to the RAM. To promote exploration and reduce the potential for anxiety associated with the maze, a first pre-exposure consisted of a 5-min exposure to the RAM with a few sugar pellets spread randomly throughout the maze. The next day, during the second pre-exposure to the RAM, sugar pellets were added only at the end of the arms to encourage head entries. Mice were taken out of the RAM when one of three possibilities was fulfilled: (1) the mouse had head entries into all eight arms within 5 min, (2) the mouse had head entries into six out of eight arms within 5–10 min, or (3) 10 min passed without six out of eight head entries. Over the 10-day testing period, each mouse received two trials per day (one 2-arm and one 4-arm separation trial) of pseudo-randomly presented combinations of the start, sample, and correct arms. A trial consisted of a sample phase with two open arms: the start arm and the pellet-containing sample arm. This was followed by a 1-min delay (time necessary to clean the maze) and a choice phase with three open arms: the start arm, the originally baited sample arm (now unrewarded), and the choice arm (now rewarded with a sugar pellet) (**Figures 1E,J**). The distance between arms was varied during testing. Spatial cues were present on the four walls surrounding the RAM for orientation during the testing phase. Mice were allowed to self-correct following entries into the originally baited arm.

#### Novel Object Recognition (NOR)

This test capitalizes on an animal’s innate preference for novelty and assesses object recognition memory (Hammond et al., 2004; Reger et al., 2009; Antunes and Biala, 2012). The delayed version of NOR (more than 10 min after familiarization) is hippocampus-dependent (Cohen and Stackman, 2015). NOR was conducted in a 46 cm by 46 cm by 52 cm gray opaque box (i.e., arena). Mouse behavior was recorded with an overhead video camera that was interfaced with a video tracking system (EthoVision XT8, Noldus). During an initial habituation phase, each mouse was placed in the empty test box for 10 min to allow exploration of the arena and diminish anxiety during the subsequent testing phases. The next day, a baseline test was conducted in which each mouse was first given a 5-min exposure to the arena to assess for pre-existing quadrant preferences. This was followed by a familiarization phase, in which each mouse was given 5 min to explore two identical objects. Short-term memory was assessed 1 h later by placing the mouse back in the arena with one familiar object and one novel object. Finally, long-term memory was assessed 24 h later when the mouse was placed in the arena with one familiar object (the previous novel object) and a new novel object (**Figure 1O**). Objects consisted of candle holders (either red or silver) and hydrogen peroxide bottles. These objects were chosen for their similarities in size (approximately 15 cm high by 6 cm wide), weight (mouse is unable to knock it over – no risk for injury) and brightness yet variability in tactile surface. The location of the objects was kept constant between trials and mice. Mice began trials at the center of the arena with their backs facing the objects. Exploration time was defined as time spent with the head oriented toward and within 2 cm of the object. After each session, the arena and objects were cleaned thoroughly with 70% ethanol to eliminate possible odor cues. Furthermore, all objects in the familiarization phase were exchanged with identical replicas during the short-term and long-term tests.

### RNA-Sequencing (RNA-Seq) and Bio-informatics

RNA-Seq was performed using samples from mice housed for 4 weeks in the LD, RUN, and CE enrichment paradigms. Notably, mice used for RNA-Seq studies were a separate cohort from those used in behavioral studies, avoiding the possibility that gene expression measurements are altered by a previous learning experience. To promote RNA preservation during the DG microdissection procedure, mice were injected with a lethal dose of Xylazine (0.1%) and Chloral hydrate (7%), then perfused trans-cardially with PBS containing sodium fluoride (50 mM), sodium pyrophosphate (1 mM) and SigmaFAST protease inhibitors (Sigma). RNA was isolated from snap-frozen microdissections of the entire DG using the RNeasy mini microarray kit as per manufacturer’s instructions (Qiagen). In addition to the three principle groups (LD, RUN, CE), L-RUN and H-RUN groups were generated by pooling RNA from the mice with the four lowest and four highest running distances, yielding a total of five groups. Sequenced mRNA fragments were trimmed for adapter sequences and then mapped to the reference mouse genome assembly version mm10 using Tophat (version 2.0.10) (Trapnell et al., 2009). Gene expressions were then estimated by using the HTSeq tool to compute read counts on RefSeq genes (Anders et al., 2015). For exploratory purposes, DESeq2 was used to normalize read counts, extract regularized log values and compute log fold changes (Love et al., 2014). Differentially expressed genes that were up- or down-regulated by a factor of log2(0.3) or more were used to investigate enriched pathways and functions through the use of QIAGEN’s Ingenuity Pathway Analysis (IPA^®^, QIAGEN Redwood City^1^). All samples were normalized at once, allowing comparison between samples in the form of principal component analysis (PCA) and hierarchical clustering. Genes were sorted according to their absolute fold change, and only genes with a log fold change ≥ 0.3 or ≤-0.3 were analyzed further. PANTHER was used to know in what main biological process categories the 380 significantly changed genes from the heatmap are found. Venny2.1.0 was used to generate the list of gene expression changes specific to each group (see Supplementary Materials). Gene lists were examined using Enrichr (Chen et al., 2013; Kuleshov et al., 2016) to investigate the Jaspar and Transfac transcription factor binding sites, GO biological processes, GO cellular components, GO molecular functions, KEGG and REACTOME pathways that were modulated.

### RT-Quantitative PCR (RTqPCR)

qPCR primers for each tested gene were designed using the Universal Probe Library tool from Roche. Total RNA was extracted from dissected tissue using RNAsolv reagent (Omega Biotek) and chloroform followed by isopropanol precipitation. Reverse transcription was performed from 1 μg of total RNA using the superscript VILO reverse transcription mix (Invitrogen). Quantitative PCR was performed on 2 μl of 1:10-diluted cDNA using SYBR Green I master (Roche) on a LightCycler 480 thermocycler. polr2d gene was used as a reference gene for ddCt quantification.

### Immunohistochemistry

Mice used for behavioral testing were processed for immunohistochemical analyses. Mice received a lethal dose of ketamine (Bimeda-MTC), xylazine (Bayer Healthcare), and acepromazine (Boehringer Ingelheim Canada, Ltd.) and were perfused transcardially with phosphate-buffered saline (PBS), followed by 4% formaldehyde (pH 7.4) that was freshly prepared from paraformaldehyde (Fisher). Brains were then removed, further post-fixed in 4% formaldehyde overnight, and serially sectioned at 40 μm thickness using a vibrating microtome (Leica VT1000S, Leica Microsystems, Richmond Hill, ON, Canada). Sections were stored at -20°C in an antifreeze solution (glycerol:ethylene glycol:PBS 1X, 3:3:4) until further use.

Immunohistochemical procedures were performed as detailed previously (Gregoire et al., 2014). Goat anti-rabbit NeuroD was used at 1:500 (Santa Cruz Biotechnology). Microscopy was performed using a motorized Olympus IX81 microscope (40X objective). As described previously (Gregoire et al., 2014), the number of cells positive for NeuroD in the subgranular zone (SGZ)/granular zone (GZ) was quantified on every 6th section between Bregma -1.06 and -2.98 mm of the hippocampus (8 sections/marker/animal). The raw cell counts were corrected for oversampling due to split cells by multiplying by (1 – object height/section height), where the object height refers to the average diameter of the marker in question. To calculate the mean density of marker-positive cells, the corresponding SGZ/GZ reference volumes of the sections were determined using the Cavalieri principle (grid size of 10 microns, 20X objective) in StereoInvestigator (MBF Bioscience, VT); mean cell density was then obtained by dividing the corrected total number of marker-positive cells on the sampled sections by the sum of the section SGZ/GZ reference volumes. Results are expressed as mean number of marker-positive cells per mm^3^ of SGZ/GZ. All quantifications were performed on coded slides by an experimenter blinded to sample identity.

### Statistical Analyses

For immunohistochemistry experiments, all experimental groups were analyzed by one-way ANOVA and a Dunnett’s multiple comparison test that compares the mean of each experimental group with the mean of the control group (i.e., LD group). For the RAM experiment, a two-way ANOVA was used to assess the effect of the groups and trials on the dependent variables of (i) % of time in newly baited arm, and (ii) entries into previously baited arm. A one-way ANOVA was also used to test for group differences in the 10-day means of these dependent variables. For the NOR task, a two-way ANOVA was used to assess the effect of the groups and object novelty (time spent around familiar vs. novel objects) on the preference ratio. A one-sample *t*-test was also performed to detect performance above chance (preference ratio of 0.5). For both RAM and NOR experiments, either a Dunnett’s or Tukey’s multiple comparison test was performed only when a significant effect was observed following the two-way ANOVA analysis. For all the experiments, significance level was set at α = 0.05. Error bars represent standard error of the mean.

## Results

### Learning and Memory Differences in Running Versus Socially Enriched Mice

Since mouse strain can strongly influence the magnitude of EE effects on the hippocampus (Clark et al., 2011), we specifically used CD1 mice for these experiments. CD1 mice are an outbred strain that exhibits robust increases in DG proliferation and neurogenesis when housed individually with a voluntary running disk for 4 weeks (Bednarczyk et al., 2009, 2011; Gregoire et al., 2014). Based on this, we first sought to establish whether runners in such a paradigm (i.e., CD1 mice with at least 4 weeks of running) would show detectable differences in tests of hippocampus-mediated functions.

Adult male CD1 mice were housed individually with running disks (RUN group) and were compared with mice housed individually with a locked disk (LD group) or in social groups of three with a locked disk (SOC group) (**Figure 1A**). Following 41 days of continuous housing in RUN, LD, and SOC conditions, mice were then subjected to an additional 2 weeks of 8-arm radial arm maze (RAM), and NOR paradigms (**Figure 1B**). 6 weeks of enrichment were provided prior to behavioral testing to allow sufficient time for integration of newly generated neurons, and the respective housing conditions were maintained during the additional testing period to avoid introduction of additional variables. The average running distance for the RUN mice was 9.88 ± 0.42 km/day, with the bottom half and top half running 8.65 ± 0.41 and 11.12 ± 0.26 km/day, respectively. Notably, RUN, LD, and SOC mice did not exhibit differences in baseline locomotion that might affect performance in these behavioral tests (**Figure 1C**), and post-testing assessment of neurogenesis confirmed that the RUN group had increased numbers of neuroblasts after 8 weeks of EE (**Figure 1D**), as reported previously after 4 weeks (Bednarczyk et al., 2009, 2011; Gregoire et al., 2014).

RAM is an incentive-driven test of spatial learning and memory. Mice were exposed to two testing periods daily for 10 days, including both a 2-arm (90°) and a 4-arm (180°) configuration each day (**Figures 1E,J**) (see section “Materials and Methods”). *Time in newly baited arm* is a measure of cognitive flexibility (i.e., the ability to abandon a previous association and establish a new one) (**Figures 1F–L**). When a two-way ANOVA was used to assess the effects of experimental group and trial # on cognitive flexibility, no interaction or significant effects of these independent variables was detected in either the 2- or 4-arm variations, although multiple comparisons showed that only the SOC group showed a tendency for an initial increase in the 2-arm variation (SOC: *p* = 0.0675 vs. LD, trial 1) (**Figure 1F**). *Entries into previously baited arm* is a measure of spatial memory recall or permanence (**Figures 1H–N**). No interaction effect was observed for experimental group and trial # on spatial memory in either the 2-or 4-arm variations. However, there were significant individual effects of group and trial # in both variations. In the 2-arm variation (**Figure 1H**), both the group effect [*F*(2,227) = 3.178, *p* = 0.0435] and the trial # effect [*F*(9,227) = 1.998, *p* = 0.0405] were significant. In the 4-arm variation (**Figure 1M**), the group effect was very significant [*F*(2,230) = 5.822, *p* = 0.0034] and the trial # effect was extremely significant [*F*(9,230) = 3.552, *p* = 0.0004]. Comparison of the mean scores over the 10 days of trials revealed that the improved spatial memory seen in the RUN group was driven specifically by the performance of the high runners (**Figures 1I,N**).

Novel object recognition is a test of object memory that is incentive-independent, capitalizing on an innate preference for novelty. Object memory retention was assessed at short-term (1 h) and long-term (24 h) intervals (**Figure 1O**) and the proportion of time spent with the novel object was expressed as a preference ratio at each interval (**Figure 1P**). When a two-way ANOVA was used to assess the influence of experimental group and object novelty on preference ratio, there was no interaction effect and no effect of the experimental group in either the short-term or long-term paradigms; thus, RUN, LD, and SOC groups did not perform significantly differently from each other. Although there was an extremely significant effect of object novelty in both paradigms [short-term: *F*(3,123) = 76.17, *p* < 0.0001, long-term: *F*(5,205) = 45.37, *p* < 0.0001], the preference ratio never exceeded 0.6, indicating that the mice exhibited highly consistent, but relatively weak, NOR. We therefore used a one-sample *t*-test to ask whether individual groups show a performance above chance (i.e., above a preference ratio of 0.5). At the 1 h time-point, the SOC group showed a statistically significant performance above chance and the LD group nearly reached significance (LD, *p* = 0.0506; SOC, *p* = 0.0125; RUN, *p* = 0.7442). At the 24 h time-point, no groups were significantly above chance, but only the RUN group approached statistical significance (LD, *p* = 0.9528; SOC, *p* = 0.2665; RUN, *p* = 0.0770). Thus, while no groups showed a statistically significant change in NOR, it is possible that the NOR test parameters were insufficiently robust to resolve differences between the housing paradigms.

Overall, we conclude from these behavioral data that RUN mice, specifically the high-distance runners, exhibit detectable performance differences in the hippocampus-regulated RAM test, changes consistent with improved spatial memory.

### Design and Validation of the RNA-Seq Transcriptomics Paradigm

**FIGURE 2:**
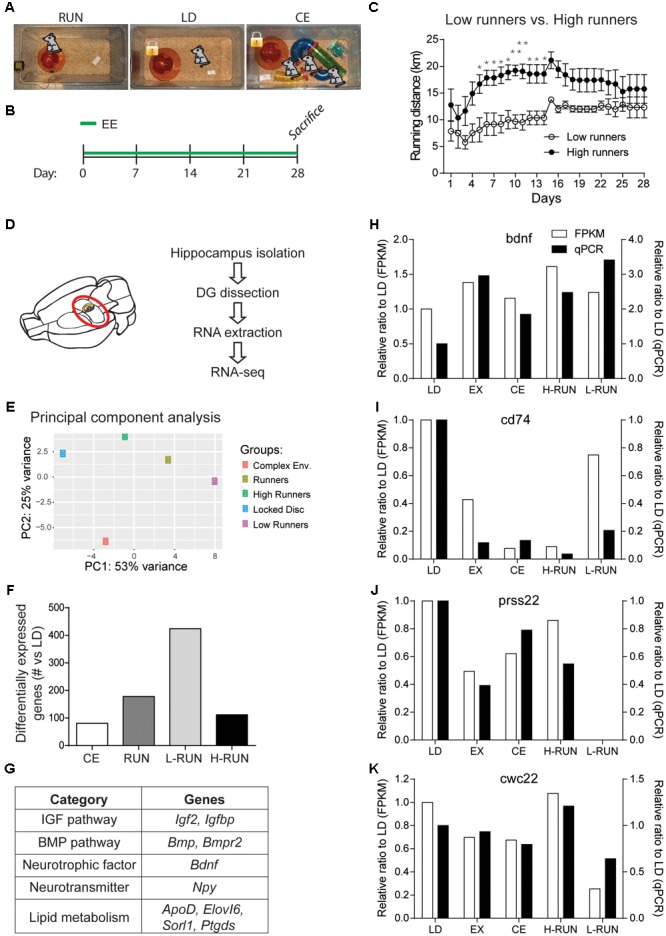
Design and validation of the RNA-Seq paradigm. **(A)** EE groups. **(B)** Timeline of experiment. **(C)** Daily running distances of the low runners and high runners. **(D)** Schematic of the RNA-Seq experiment. **(E)** Principal component analysis (PCA) of the top 5000 varying genes. Two components were sufficient to separate the five conditions. **(F)** Number of significantly modulated genes per group (vs. LD) at a cut-off of log2(0.3). **(G)** Table of several known running-induced genes that are significantly modulated in one or more of the running groups. Comparison of the changes measured by RNA-Seq versus qPCR for four genes, BDNF **(H)**, CD74 **(I)**, Prss22 **(J)**, and Cwc22 **(K)**. RNA used for the qPCR measurements was obtained from the contralateral DG of the animals used for RNA-Seq. ^∗^*P* ≤ 0.05; ^∗∗^*P* ≤ 0.01.

To investigate how running affects hippocampal function, we focused on the DG and used RNA-Seq to perform an unbiased assessment of genome-wide changes in DG gene expression. New groups of RUN, LD, and running-independent EE mice were established. For the latter group, since SOC mice did not exhibit detectable differences in our behavioral tests, we used a more enriched running-independent CE comprised of social-enrichment, a locked disk, and tunnels (**Figures 2A,B**). After 4 weeks, the DG were micro-dissected, and the RNA extracted from each sample individually (**Figure 2D**). Since CD1 mice are an outbred strain, we combined equal amounts of RNA from a large number of samples (*N* = 8/group) to generate pooled samples from each of the LD, RUN, and CE groups. In addition to the three main experimental groups, RNA of mice in the RUN group were also used to create high run (H-RUN) and low run (L-RUN) groups (*N* = 4/group) based on individual running distances. H-RUN and L-RUN mice ran averages of 17.00 ± 0.45 and 10.65 ± 0.39 km/day, respectively (**Figure 2C**); notably, the L-RUN group ran approximately the same as the high-runners in the behavioral experiments. RNA samples were used for library preparation, RNA-sequencing, sequence alignment, and transcript quantification as described (see section “Materials and Methods”). RNA-Seq data are deposited in the Gene Expression Omnibus, GEO accession #GSE107356.

To gain an initial overview of the EE-induced transcriptomic changes, we performed a PCA of the 5000 most varying genes (**Figure 2E**). PCA showed that, compared to the LD control, the three RUN groups varied along PC1 and the CE group varied along PC2; thus, there are differences in the global gene expression patterns between the running and running-independent EE paradigms. To begin assessing the quality of these RNA-Seq data, we then generated lists of all genes that were up- or down-regulated by at least log2(0.3) (*p* ≤ 0.05) for each group versus the LD control group, yielding 81 genes for CE, 178 for RUN, 112 for H-RUN, and 424 for L-RUN (**Figure 2F**). Several lines of evidence supported the validity of this data. First, many genes and pathways previously implicated in the hippocampal effects of running were successfully detected, including the IGF and BMP pathways, BDNF, NPY, and lipid metabolism genes (**Figure 2G**). Second, that substantially more significant changes in gene expression were detected in the L-RUN than the H-RUN group is consistent with a previous microarray analysis of the entire hippocampus in rats exposed to mild versus intense treadmill running (Inoue et al., 2015). Third, we also isolated RNA from the contralateral DG of the same animals and performed qPCR to confirm a number of the changes identified by RNA-Seq (**Figures 2H–K**). This included genes that were either known (*Bdnf*) or previously unknown (*CD74, Prss22 Cwc22*) in the context of EE. Using these separate RNA samples and alternate qPCR approach, we could confirm our transcriptomic data showing that (i) BDNF mRNA is increased in all groups of running mice, (ii) CD74 mRNA is decreased in all EE groups relative to LD controls, (iii) Cwc22 mRNA is markedly decreased in L-RUN mice, and (iv) Prss22 mRNA virtually disappears in L-RUN mice. Together, these confirmations supported the validity of the data obtained from our RNA-Seq experiment.

### Major Classes of EE-Induced Transcriptomic Changes

**FIGURE 3:**
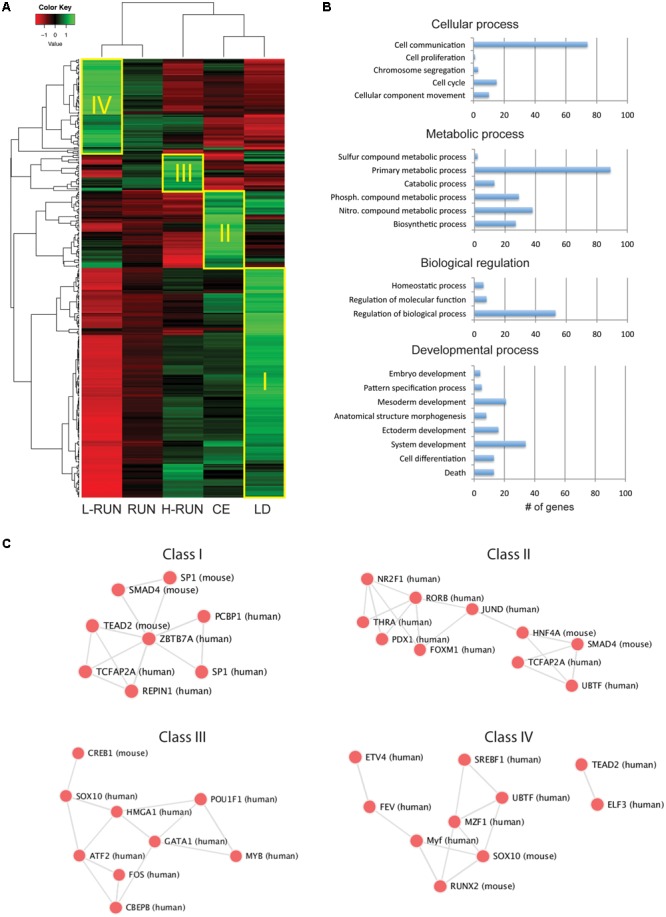
Overview of EE-induced transcriptomic changes. **(A)** Z-score heatmap of the 380 genes having at least a 1.4-fold difference across any two groups. Hierarchical clustering identifies four large classes of co-regulated genes (I–IV), which are preferentially expressed in the LD (Class I), CE (Class II), H-RUN (Class III), or L-RUN (Class IV) experimental groups. **(B)** The top four GO Biological Processes represented within the above 380 genes. Shown are the number of genes found within each sub-category of the top four GO Biological Processes. **(C)** Transcription factor networks associated with the Classes I–IV groups of co-regulated genes.

To visualize the EE-modulated gene expression patterns, genes modulated at least 1.4-fold across any two groups were hierarchically clustered and used to generate a Z-score heatmap (**Figure 3A**). As expected, the RUN group clustered intermediate to its constituent H-RUN and L-RUN groups. Notably, it was the CE group that clustered closest to the LD control group, and of the RUN/H-RUN/L-RUN running groups, it was the H-RUN group that was most similar to the CE and LD groups. Gene Ontology (GO) analysis of these 380 differentially expressed genes showed they were most enriched in the GO Biological Process categories of Cellular process (50.1% of genes), Metabolic process (31.3% of genes), Biological regulation (18.1% of genes), and Developmental process (17.5% of genes) (**Figure 3B**).

The heatmap revealed the presence of several large classes of co-regulated genes, which we classified as being preferentially expressed in the LD controls (Class I, 198 genes), CE group (Class II, 68 genes), H-RUN group (Class III, 32 genes), or L-RUN group (Class IV, 82 genes) (**Figure 3A**). Interestingly, transcriptional analysis showed that the genes in each class are regulated by distinct transcription factor networks, revealing the presence of EE-specific transcriptional programs (**Figure 3C**). Thus, it appears that some transcription factor networks are suppressed in both running and running-independent EE paradigms (Class I), some are activated primarily in running-independent EE (Class II), and within the running group, some are preferentially activated in either high runners (Class III) or low runners (Class IV) but not both. Based on these observations, we proceeded to deeper bio-informatics comparisons between specific EE groups.

### Running vs. Running-Independent EE: RUN Modulates Glutamatergic Synapse, Cell Growth, and Growth Factor Signaling Genes While CE Affects Extracellular Matrix Genes

**FIGURE 4:**
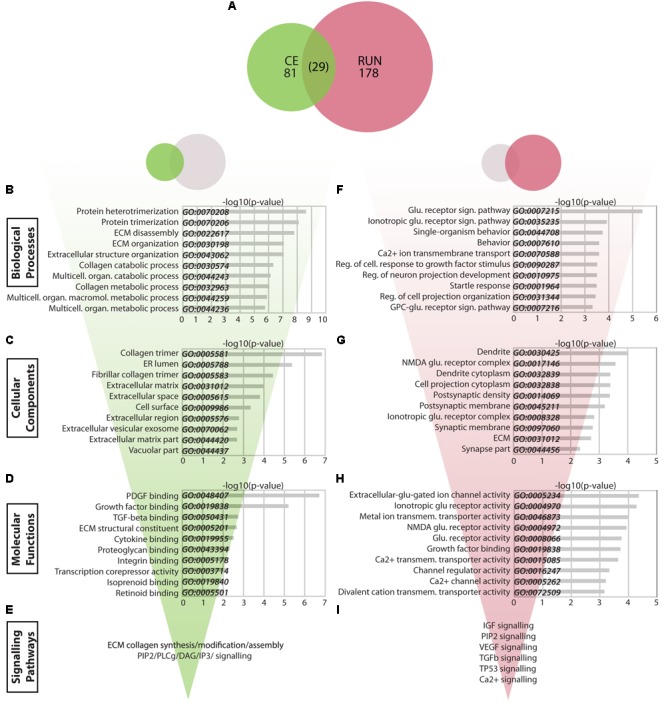
Transcriptomic changes in the CE vs. RUN groups. **(A)** Venn diagram showing the relationship between significantly modulated genes in the CE and RUN groups. **(B–I)** Bio-informatic analyses of modulated genes in the CE group **(B–E)** and RUN group **(F–I)** were performed by gene set enrichment analysis for GO Biological Processes **(B,F)**, GO Cellular Components **(C,G)**, GO Molecular Functions **(D,H)**, and KEGG and Reactome pathways **(E,I)**. For each of the GO categories, the top 10 enriched gene sets are shown plotted against their –log10(p-value). Supplementary Table S1: Spreadsheet of the most modulated genes for the CE environment versus the LD control. Supplementary Table S2: Spreadsheet of the most modulated genes for the RUN environment versus the LD control.

Using the lists of genes that were altered in EE mice versus the LD controls (**Figure 2F** and Supplementary Tables S1, S2 Fig.4-1, and Fig. 4-2), we first compared the gene expression changes in the RUN (178 genes) and CE (81 genes) enriched environment mice. Interestingly, of the 259 total changes in these two groups, only 27 (10.4%) occurred in both groups (**Figure 4A**). To gain insight into the cellular and molecular pathways affected by these gene expression changes, we then used EnrichR to perform GO analyses (GO 2015 Biological Process, Cellular Component, and Molecular Function) and further pathway analyses (KEGG 2016 and Reactome 2016 databases).

The 81 CE-associated genes showed a particularly strong enrichment for GO Biological Process gene sets related to the extracellular matrix (ECM) (**Figure 4B**). Genes within the top 30 enriched gene sets clustered into four main categories: ECM (*Timp2, Dcn, Ttr, Col1A2, Col1A1, Col6A2, Col6A1, Col5A1, Col23A1, Sulf2, Thbs1*), prostanglandin/prostanoids (*Mif, Cd74, Ptgds*), FGF and WNT signaling (*Ppp1cb, Cd109, Skil, Wnt4, Sulf2, Thbs1*), and negative regulation of signal transduction (*Prkcd, Itpr1, Sca1, Gstp1, Mif, Cd74, Thbs1, Timp2*). The modulated genes produce proteins that localize mainly to the ECM/cell surface (GO Cellular Components, **Figure 4C**) and that are implicated in binding of extracellular molecules such as growth factors, ECM constituents, hormones, and others (GO Molecular Functions, **Figure 4D**). Consistent with this, KEGG pathway analysis identified two main gene clusters, which were enriched in data sets for either ECM-receptor interactions, protein digestion and focal adhesion (*Col1A1, Col1A2, Col6A1, Col6A2, Thbs1, Col5A1, PPP1CB*) or for inflammatory processes, proteoglycans, long-term potentiation (LTP), and vascular smooth muscle cells (*Itpr1, Adcy1, Ppp1cb, Grin2a, Prkcd, Htr2c, Rasgrp1*). Specific cellular pathways identified by Reactome were synthesis/modification/assembly of ECM collagen and PIP2/PLCg/DAG/IP3 signaling (*p* < 0.005) (**Figure 4E**).

The 178 significantly changed genes in the RUN group differ strikingly from those in the CE group and are particularly enriched in GO Biological Process gene sets related to glutamate signaling and cell growth (**Figure 4F**). Genes enriched within the top 30 gene sets cluster into four groups, which are associated with glutamate receptor signaling (*Grik3, Trpm3, Grin2b, Grin2a, Grin3a, Homer2*), cellular growth/projection development (*Sema4d, Rap1gap2, Lzts1, Dpysl3, Cdkl5, Bmpr2, Skil, Tnr, Mef2a, Cpeb3, Tenm1, Tgfbr1*), regulation of responses to growth factors (*Ubb, Prkcb, Htra4, Glg1, Flt1, Ccbe1, Tgfbr1, Skil*), and behavior (*Grin2a, Grin2b, Ptgds, Strn, Homer2, Penk, Arc, Etv1, Igf2, Kcnip3, Sgk1, Cpeb3, Mef2a, Tnr*). The main locations of these gene products are synapses and dendrites (*Grin2a/2b/3a, Grik3, Strn, Lzts1, Arc, Homer2, Mib1, Itpr1, Kcnip3, Ddn, Cpeb3, Bmpr2, Apod, Uhmk1, Cdkl*) or the ECM (*Tnr, Spock2, Mgp, Matn2, Glg1, Fmod, Ecm2, Col1a2, Ccbe1, Aebp1*) (GO Cellular Components, **Figure 4G**), where they are involved in glutamate receptor activity and membrane transport for metal ions and calcium (*p* < 0.0005) (GO Molecular Functions, **Figure 4H**). RUN genes are enriched in KEGG data sets associated with glutamatergic synapses, phosphatidylinositol signaling, calcium signaling, circadian entrainment, LTP, and cGMP-pKG signaling (*p* < 0.005). Using Reactome, more specific pathways were revealed that included IGF (*Pappa, Igfbp6, Igfbp5, Igf2*), PIP2 (*Dgki, Dgkh, Itpr1*), VEGF (*Akt3, Ubb, Ksr2, Irs2, Flt1, Itpr1, Prkcb, Grin2b, Grin2a*), TGFb (*Ubb, Tgfbr1, Cbl, Skil*), and TP53 (*Sgk1, Akt3, Ubb*) (*p* < 0.0005) (**Figure 4I**).

Overall, these analyses reveal that running and running-independent EE have consequences on distinct cellular and molecular targets, with CE primarily affecting ECM interactions and RUN mainly impacting glutamatergic synapses, cell growth, and growth factor signaling.

### “Core” Running Changes Involve Glutamate and IGF Signaling

**FIGURE 5:**
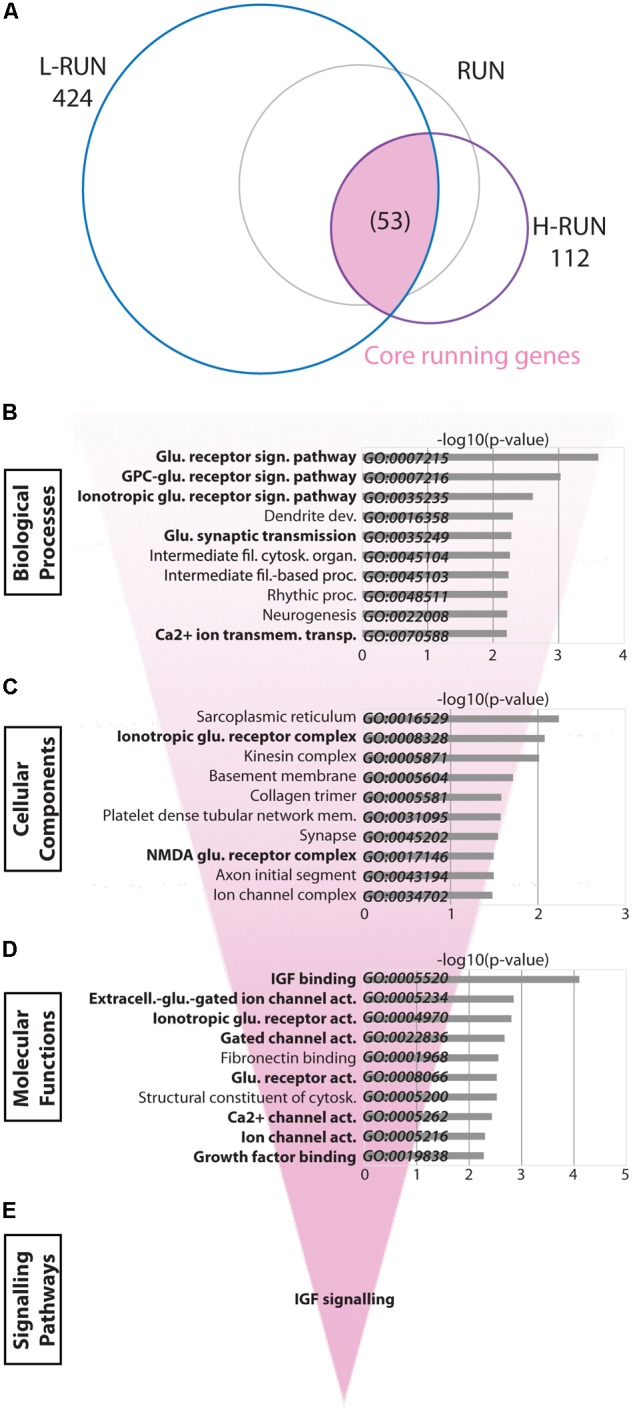
“Core” running-induced transcriptomic changes. **(A)** Venn diagram showing the relationship between significantly modulated genes in the L-RUN and H-RUN groups, focusing on the 53 “Core” running genes that were modulated in both groups (highlighted). Bio-informatic analyses of Core running genes was performed by gene set enrichment analysis for GO Biological Processes **(B)**, GO Cellular Components **(C)**, GO Molecular Functions **(D)**, and KEGG and Reactome pathways **(E)**. For each of the GO categories, the top 10 enriched gene sets are shown plotted against their –log10(p-value). Gene sets in bold had likewise shown enrichment for genes modulated in the RUN group (**Figure 4**).

We next investigated the transcriptional consequences of running in greater detail. The RUN group is comprised of samples from both the L-RUN and H-RUN groups, which ran 10.65 and 17.00 km/day respectively and exhibited considerable differences in the number of significantly altered genes (424 and 112 genes, respectively) (**Figure 5A**). To identify the “core” processes associated with running, we analyzed the subset of 53 genes whose expression was altered in both L-RUN and H-RUN groups. Similar to the overall RUN group, GO Biological Process analysis of the core running genes prominently highlighted glutamate neurotransmission, with five of the top 10 gene sets related to glutamate receptors and glutamate signaling (**Figure 5B**). Consistent with this, GO Cellular Component analysis included gene sets for Ionotropic glutamate receptor complex and NMDA glutamate receptor complex (**Figure 5C**), and GO Molecular Functions further identified several glutamate signaling gene sets (**Figure 5D**). In addition to glutamate neurotransmission, IGF binding/signaling was also significantly implicated by GO Molecular Functions (*p* < 0.0005) and Reactome pathway analysis (*p* < 0.005) (**Figure 5E**). Thus, glutamate neurotransmission and IGF signaling are common targets of the L-RUN and H-RUN groups.

### Low Runners Exhibit Multi-System Synaptic Changes, Extracellular Matrix Alterations, and Broad Changes in Growth Factor and Calcium Signaling

**FIGURE 6:**
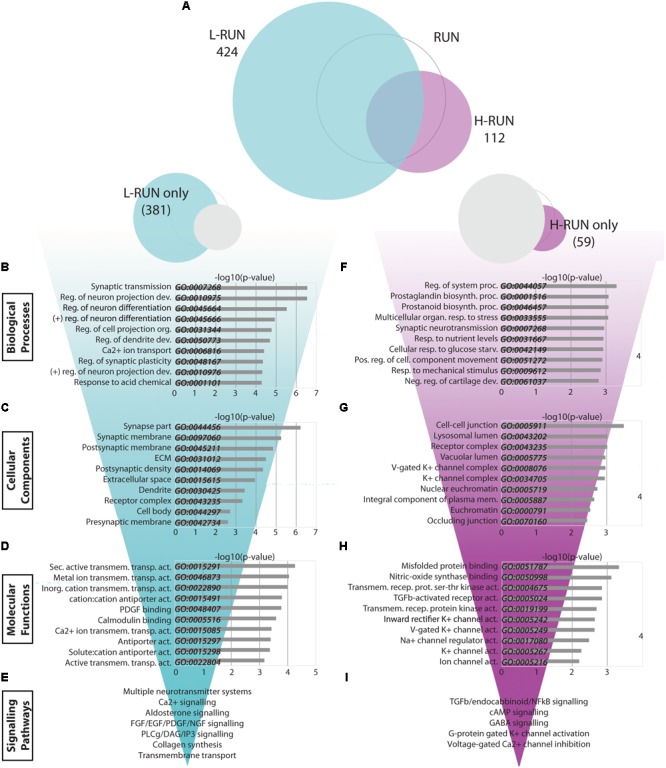
Transcriptomic changes specific to low runners and high runners. **(A)** Venn diagrams showing the relationship between significantly modulated genes in the L-RUN and H-RUN groups, focusing on the genes that were L-RUN-specific and H-RUN-specific. **(B–I)** Bio-informatic analyses of modulated genes in the L-RUN group **(B–E)** and H-RUN group **(F–I)** were performed by gene set enrichment analysis for GO Biological Processes **(B,F)**, GO Cellular Components **(C,G)**, GO Molecular Functions **(D,H)**, and KEGG and Reactome pathways **(E,I)**. For each of the GO categories, the top 10 enriched gene sets are shown plotted against their –log10(p-value). Supplementary Table S3: Spreadsheet of the most modulated genes for the L-RUN environment versus the LD control. Supplementary Table S4: Spreadsheet of the most modulated genes for the H-RUN environment versus the LD control.

Comparison of the L-RUN and H-RUN groups showed that 371 gene expression changes were L-RUN-specific (87.5% of the L-RUN-associated genes) (**Figure 6A**, Fig.6-1, and Fig. 6-2). L-RUN-specific genes were mainly enriched in GO Biological Process gene sets associated with either synaptic transmission/plasticity or neuron projection/differentiation/dendrite development (**Figure 6B**). The L-RUN-specific gene products show GO Cellular Component enrichment at synapses, dendrites, and the extracellular space (*p* < 0.00005) (**Figure 6C**), and are implicated in various types of transmembrane transporter activity, collagen-associated growth factor-binding, and calcium/calmodulin binding (*p* < 0.0005) (GO Molecular Functions, **Figure 6D**). KEGG pathway analysis revealed that L-RUN-specific changes are associated with alterations in ECM interactions and with broad changes in neurotransmission that include cholinergic, dopaminergic, glutamatergic, and gabaergic synapses (*p* < 0.005). Additional gene set enrichments include calcium signaling, circadian entrainment, aldosterone signaling and longevity pathways (*p* < 0.005). Using Reactome, signaling changes that can be identified that include general neurotransmission, collagen synthesis, FGF/EGF/PDGF/NGF signaling, PLCg/DAG/IP3 signaling, calcium/calmodulin signaling, and transmembrane transport (*p* < 0.005) (**Figure 6E**).

Overall, the abundant changes in gene expression that are L-RUN-specific involve multiple neurotransmitter systems, ECM interactions, several growth factor families, and calcium-mediated signaling pathways.

### High Runners Show Transcriptomic Changes Associated With Stress Responses and Negative Regulation of Synaptic Activity

Surprisingly, H-RUN mice exhibited fewer and distinct transcriptomic changes versus L-RUN mice. To better understand this, we performed a bio-informatics analysis of the subset of 59 H-RUN-specific gene expression changes. Interrogation of the GO databases revealed H-RUN-specific Biological Processes related to regulation of system processes (*Kcnj2, Htr2c, Sgk1, Dsg2, Atp2b4, Ptgs2*), prostaglandin/prostanoid biosynthesis (*Ptgs2, Cd74*), organismal and nutrient stress responses (*Htr2c, Bdnf, Adcyap1r1*), and synaptic transmission (*Kcnj2, Htr2c, Nptx2, Kcnj16, Kcnf1, Gabra3*) (*p* < 0.005) (**Figure 6F**). H-RUN-specific gene products localize to cell-cell junctions, lysosome/vacuoles, receptor complexes and potassium channel complexes (GO Cellular Compartments, **Figure 6G**), and are involved in misfolded protein binding, NOS binding, TGFβ receptor activity, and potassium channel activity (GO Molecular Functions, **Figure 6H**). KEGG pathway analysis revealed H-RUN-specific enrichment for TGFβ signaling (*p* < 0.005), as well as endocannabinoid, cAMP, and NFκB signaling (*p* < 0.05). Interestingly, Reactome analysis further linked these changes to activation of GABA receptors and G-protein gated potassium channels and to inhibition of voltage-gated calcium channels (*p* < 0.005) (**Figure 6I**), changes predicted to suppress neurotransmission.

Given the relatively small number of H-RUN-specific genes, we also performed a bio-informatics analysis of the 112 changes within the complete H-RUN data set. This showed similar overall enrichments to the 59 H-RUN-specific changes, with additional highlighting of GO Biological Processes associated with stress, including multicellular organismal response to stress (*p* < 0.00005), negative regulation of synaptic transmission, behavioral fear/defense responses (*p* < 0.0005), and negative regulation of homeostatic process (*p* < 0.005). Collectively, these data reveal that the H-RUN group exhibits elevated organismal and cellular stress responses, distinct patterns of signaling changes, and a predicted GABA- and potassium-mediated suppression of synaptic activity.

## Discussion

The present study explores the transcriptional mechanisms underlying EE-induced changes in hippocampal function. Running and running-independent EE can have differential effects on hippocampal structure and function, and despite using outbred CD1 mice, we indeed detected running-specific behavioral differences that are consistent with improved spatial memory. Focusing on the DG, we then used RNA-Seq to identify genome-wide gene expression changes in response to running versus running-independent EE. Gene expression profiling using RNA-Seq is a powerful tool for comprehensively studying cellular responses to physiological or experimental stimuli, as this technology is unbiased (does not rely on the existence of pre-existing probes) and offers a broad dynamic range that is not affected by hybridization sensitivity (for low abundance transcripts) or signal saturation (for high abundance species). As discussed below, bio-informatics analyses reveal that transcriptional changes in the DG are highly influenced by both the nature of the EE (running vs. running-independent) and, in the case of runners, the degree of running (low- vs. high-distance runners). Moreover, our RNA-Seq data identifies a wide range of novel actors that can be explored as potential mediators of EE effects on brain function.

### Running-Induced Changes in Hippocampal Function

We used two behavioral tests to assess changes in hippocampus-regulated functions, RAM (for spatial learning and memory) and NOR (for object memory). In the RAM, the RUN group showed significant differences in performance that are consistent with improved spatial memory, and that were largely driven by the performance of the highest distance runners. In the NOR, there was no significant group-wise difference in performance. With regard to the latter, although the mice did indeed show statistically significant NOR, analysis of the data suggests that test parameters were not optimal for detecting group differences. Indeed, since only one of the three groups reached a preference ratio that was significantly above chance even in the 1 h short-term memory paradigm, we cannot exclude that further optimization of test parameters would reveal group differences. Measuring differences in cognitive function with behavioral tests is challenging and can be influenced by a wide range of genetic, environmental, and experimental parameters (Van Meer and Raber, 2005). Moreover, the greater inter-animal variability of outbred CD1 mice increases the likelihood that potential findings are physiologically relevant to the general population, but hinders achieving statistically significant differences between groups; inbred lines, on the other hand, facilitate achieving statistical significance but can be considered an unusual genetic state (Svenson et al., 2012).

Interestingly, mice in the bottom half and upper half of runners in the behavioral studies (8.6 vs. 11 km/day, respectively) showed a statistically significant difference in memory in at least the 4-arm variation of RAM (**Figure 1N**), suggesting that this 30% running distance difference is functionally meaningful. Running distance has been shown to have an impact on hippocampal neurogenesis (Rhodes et al., 2003), with a positive correlation observed between running distance and new neurons in normally bred mice. However, in that study, mice selectively bred for high levels of running showed no such correlation, suggesting a possible ceiling effect. Moreover, spatial learning was improved in the normal mice, but not in the high-runners, suggesting that the running distance can have an impact on behavioral outcomes and neurogenesis (Rhodes et al., 2003).

### Running-Induced Transcriptional Changes in the DG

RNA-Seq was used to probe the gene expression changes associated with running versus running-independent EE. These transcriptomic experiments were performed using a separate cohort of mice that was not behaviorally tested, avoiding the possible confounding effects of the learning and memory tests on DG gene expression. The transcriptomic changes caused by running prominently affected genes whose products localize to synapses and dendrites, with notable enrichments in categories related to glutamate receptor signaling, cell growth/projection development, and regulation of responses to growth factors.

In the case of glutamate receptor signaling, recent studies using trans-synaptic retrograde tracing and *in vivo* imaging approaches have revealed that running EE markedly increases the pattern of inputs into the DG from the entorhinal cortex, from other hippocampal subfields, and from sub-cortical structures (Bergami et al., 2015; Vivar et al., 2016). Consistent with such connectivity changes, we found that running prominently enriched glutamate signaling-associated GO gene sets. Genes implicated within these GO categories are involved with glutamate receptors and/or their downstream signaling events, and potentially represent key mediators of the glutamatergic alterations. This includes subunits for NMDA receptors (*Grin2b, Grin2a, Grin3a*) and kainate receptors (*Grik3*), regulators of metabotrophic receptors (*Homer2*), calcium-related channels and transporters (*Trpm3, Cacna1e, Atp2b4*), the calcium-regulating IP3 receptor (*Itpr1*), the calcium and DAG-activated kinase (*Pkcb*), and DAG kinases (*Dgk1, Dgkh*).

The categories of cell growth/projection development and regulation of responses to growth factors are biologically inter-related. Our bio-informatic analysis revealed a variety of potential mediators of cell growth/projection changes. Specific genes that were implicated affect Semaphorin pathways (*Sema4d, Dpysl3*), TGFβ superfamily receptors (*Tgfbr1, Bmpr2*), Tenascin pathways (*Tnr, Tenm1*), cell cycle regulators (*Cdkl5, Lzts1*), transcriptional regulators (*Mef2a, Skil*), and others. Growth factors exert powerful influences over such cell growth and development pathways, and we indeed observed running-induced growth factor changes in the IGF, BDNF, VEGF, and TGFβ pathways. These changes included genes for growth factors themselves (*Igf2, Bdnf*), growth factor binding proteins/regulators (*Igfbp6, Igfbp5, Pappa, Htra4*), growth factor receptors (*Tgfbr1, Flt1*), receptor-associated components and downstream, signaling proteins/modulators (*Akt3, Ksr2, Irs2, Itpr1, Cbl, Sgk1, Ubb, Prkcb, Grin2a, Grin2b, Skil*), including inositol/calcium signaling (*Itpkb, Prkcb, Impad1, Itpr1, Dgki, Dgkh, Atp2b4, Cacna1e*) and cGMP/PKG signaling (*Nos1, Mef2a, Mef2d*). Our data thus reveal key molecules and cascades impacted by running in the DG.

### Differences Between Low and High Runners

L-RUN and H-RUN groups showed surprisingly distinct sets of transcriptional changes, with L-RUN mice undergoing nearly fourfold more significant changes in gene expression. Analysis of the restricted group of changes shared between the L-RUN and H-RUN groups identified glutamate signaling and IGF signaling as core targets of running. By focusing on the changes in L-RUN mice, a broader and more detailed picture of the overall transcriptional changes induced by running is revealed. Like RUN mice, L-RUN mice showed alterations in genes whose products are localized principally to synapses and dendrites; however, L-RUN mice showed 25 GO Biological Process categories enriched to at least *p* < 0.0005, versus only 11 categories in the complete RUN group (not shown). L-RUN mice exhibited broader neurotransmission-associated changes that included KEGG enrichments for cholinergic, dopaminergic, glutamatergic, and gabergic systems; notable changes included receptors for GABA (*Gabbr2*) and acetylcholine (*Chrna7, Chrm3*). L-RUN mice also displayed greater numbers of gene changes related to cell growth/process development, which included growth factor families seen in the RUN group (BMPs and semaphorins) as well as families not identified in RUN or H-RUN groups (neurotrophin, netrin, slit, NRCAM, FGF). Furthermore, L-RUN mice showed significant enrichment for GO categories that did not emerge in other groups, including positive regulation of neurogenesis (GO:0050769) and blood vessel development (GO:0001568).

In contrast, the H-RUN group exhibited a dampening of the majority of L-RUN-associated gene expression changes, and clustered intermediate between L-RUN mice and the non-running LD and CE groups. In contrast to the other experimental groups, H-RUN-specific changes affected genes whose products normally localize with cell junctions, lysosomes/vacuoles, and K^+^ channels. High runners showed notable enrichments for stress-associated gene sets, such as protein misfolding, organismal response to stress, fear/defense responses, and negative regulation of synaptic transmission and homeostatic processes. Pathway analysis identified TGF-β, endocannabinoid, cAMP, and NFκB signaling pathways as possible mediators of these stress-associated responses, and identified GABA receptors, G-protein gated potassium channels, and voltage-gated calcium channels as plausible molecular targets mediating negative regulation of synaptic transmission. Importantly, because L-RUN and H-RUN groups were identified *post hoc*, causality and consequence cannot be distinguished, i.e., the observed differences between these groups might represent downstream effects of running differences, or alternatively, predisposing genetic factors that subsequently result in running differences.

It is worth noting that the L-RUN group in the RNA-Seq portion of this study (10.65 ± 0.39 km/day) ran approximately the same as the high-distance runners that displayed increased spatial memory in the behavioral portion of the study (11.12 ± 0.26 km/day). Might the patterns of transcriptional changes in H-RUN mice suggest that excess running can eventually have detrimental cognitive effects? In line with this idea, Inoue et al. (2015) recently showed that mild exercise increases neurogenesis in rats, whereas intense treadmill running did not increase neurogenesis and led to higher stress levels. Similarly, Naylor et al. (2005) observed that declines in hippocampal proliferation observed after 24 days of running were rescued by restricting the daily running distance. These data collectively suggest that stress pathways activated by high-intensity exercise may suppress many of the positive effects of running. Exercise is also normally a rewarding activity that modulates the dopaminergic mesolimbic circuitry (reward pathway that connects the ventral tegmental area to the nucleus accumbens) (Greenwood et al., 2011), and previous work indicates that mice bred to exhibit excessive running have dopaminergic abnormalities (Mathes et al., 2010). Interestingly, our RNA-Seq showed that only the running groups expressed enkephalin, an endogenous ligand for opioid receptors expressed in the mesolimbic system (Garzon and Pickel, 2002). Since heavy physical workload in humans can lead to impairments of cognitive function (Dupuy et al., 2014), it may be interesting to investigate whether “runner’s high” can have detrimental effects on cognitive function.

### A Transcriptional Signature for Running-Independent EE

Two running-independent groups were used in this study: the SOC group in the behavioral experiment (= LD+social housing) and the CE group in the RNA-seq experiment (= LD+social housing+tunnels). We used the SOC group for the initial behavioral experiments based on data from our previous study showing that SOC mice have a significant increase in DG neurons expressing c-fos (a surrogate marker for neuronal depolarization) (Gregoire et al., 2014). However, because the SOC group did not show performance differences in the behavioral tests, we used the further enriched CE group as the running-independent EE group for the subsequent RNA-seq studies. We previously reported that neither SOC nor CE mice show an increase in DG neurogenesis, that both have increased DG c-fos expression, but that only CE mice have a decrease in circulating levels of the stress hormone corticosterone (Gregoire et al., 2014). Thus, while the SOC group was not used for RNA-seq, we would predict that SOC and CE groups undergo distinct but largely overlapping transcriptional changes.

Here, we found that the CE group showed 81 significant changes in gene expression that were separate from those occurring in the running groups. The transcriptional changes in CE mice were statistically highly significant (ranging from *p* < 10^-9^ to 10^-5^), and involved ECM processes related to collagen synthesis/modification/assembly and intracellular signaling via the PIP2/PLCg/DAG/IP3 pathway.

Collagen synthesis/modification/assembly may mediate changes in DG function in at least two ways. First, ECM collagen directly activates integrin receptors. Second, ECM collagen modulates binding of growth factors, cytokines, and hormones to their respective cell surface receptors, thus affecting trophic properties of the cellular microenvironment. Interestingly, since collagen activation of integrin receptors has been shown to increase PIP2 hydrolysis in other systems (Cybulsky et al., 1993, 1996), the observed PIP2/PLCg/DAG/IP3 signaling changes may be a direct result of the ECM collagen changes. Thus, ECM collagen and PIP2/PLCg/DAG/IP3 signaling warrant deeper investigation as potential mediators of EE’s running-independent effects on hippocampal function.

### The Caveat of Social Isolation

It may be important to note that mice in the LD and RUN environments were housed individually, an abnormal social condition that is likely to have physiological consequences. Unfortunately, mouse running distances cannot be easily assessed unless they are individually housed, and as our RNA-seq data demonstrate, the running distance does indeed have a profound impact on the nature of the genetic changes occurring within the DG. Since both the RUN and LD groups were individually housed, we can be confident that the RUN vs. LD comparison provides us with an informative approximation of running’s genetic impacts; still, we cannot exclude the possibility that a subset of running-induced changes might have been either *dependent upon* or *inhibited by* coincident social isolation. In the case of the socially-housed CE group, however, an alternative interpretation of the gene expression differences in the CE vs. LD comparison is that they are due to the LD isolation rather than to the CE enrichment. We cannot exclude this possibility in the present study.

To our knowledge, this is the first study using RNA-seq to study transcriptome changes in the adult mouse DG following exposure to both running and running-independent enriched environments. In a very recent paper, Zhang et al. (2018) characterized transcription and methylation changes in the dorsal versus ventral DG of mice that had been raised in an enriched environment that included running wheels. Direct comparison of our data with that study is complicated by the fact that their mice were directly weaned into the EE during the peripubertal period, however their conclusions highlight the fact that our transcriptional changes may be differentially distributed along the dorsal–ventral axis of the DG (Zhang et al., 2018). Our data shows both similarities and differences to a previous RNA-Seq performed on mice exposed to 11 months of EE (that included running wheels), but the latter study looked at the whole-brain transcriptome rather than specifically the DG (Huttenrauch et al., 2016b). In another study, Inoue et al. (2015) have demonstrated transcriptome differences between two different exercise intensities (mild and intense), and like us, found distinct patterns of gene expression changes in low and high-distance runners. Interestingly, this included activation of stress pathways with high-intensity and high-distance exercise, but with different main classes of trancriptomic changes. This may be explained by differences in methodological parameters, as the work by Inoue et al. (2015) used treadmill running, whole rat hippocampus and microarray analysis, whereas we used voluntary running, microdissected mouse dentate gyri and RNA-seq.

### Implications for Promoting Brain Plasticity and Disease-Resilience

Physical exercise has been shown to have beneficial effects not only in healthy rodents, but also in rodent models of numerous pathological conditions. For example, in a post-traumatic stress disorder rat model, where large rats play the role of the resident aggressors and small rats play the intruders, rats that were exposed to a 2-week daily regimen of mild intensity treadmill exercise exhibited reduced anxiety- and depression-like behavior when exposed to social defeat (Kochi et al., 2017). As highlighted in a recent review, there is a need for further investigation of the effects of exercise on depression-like behavior using molecular and behavioral approaches (Mul, 2018). In Alzheimer’s disease (AD) models, EE approaches have largely focused on physical activity, which has been effective in increasing adult neurogenesis (Rodriguez et al., 2011; Marlatt et al., 2013). Interestingly, both voluntary and treadmill running regimens in AD rodent models have led to reduced tissue hallmarks of AD progression, such as Aβ40/42 levels and tau phosphorylation (Ryan and Kelly, 2016). Mechanisms of exercise effects on Aβ levels may include increased Aβ clearance from the CNS (Moore et al., 2016), a shift toward non-amyloidogenic processing of the amyloid precursor protein (Koo et al., 2017), and modulation of microglial activity (Rodriguez et al., 2015). With regard to the latter, studies in both healthy and AD mice have reported complex and sometimes opposing changes in microglial parameters across different brain regions in response to EE and/or exercise (Ehninger and Kempermann, 2003; Steiner et al., 2004; Ehninger et al., 2011; Rodriguez et al., 2015); this underscores the need for deeper investigation of how enrichment paradigms can have region-specific effects that differentially impact pro- and anti-inflammatory microglial phenotypes. Globally, however, the cellular consequences of exercise correlate with functional improvements and/or rescue of spatial learning and memory deficits in AD mouse models (Liu et al., 2011; Huttenrauch et al., 2016a; Ryan and Kelly, 2016).

In humans, physical activity is widely proposed as a low-cost, non-pharmacological approach that usually has positive cognitive outcomes and that can be personalized to the target population (Bherer et al., 2013). However, the available research tools in humans for investigating the underlying mechanisms of exercise on brain function (e.g., spatial memory) differ greatly from those used in rodent studies (Barak et al., 2015). For example, human studies often use tests that don’t rely on the hippocampus to look at hippocampus-dependent processes, such as spatial memory. Moreover, the growing trend in the medical field to “prescribe” exercise to promote healthy cognitive aging, while most likely beneficial, is currently done without a clear understanding of the exact types, volume, frequency, and intensity of exercise that would be optimal for each individual (Barha et al., 2017). A better understanding of the molecular changes that occur following different types of training, as well as how pre-existing genetic factors affect response to exercise, would be helpful. For example, midlife adults with a Val66Met polymorphism in the BDNF gene are less responsive to aerobic training and demonstrate reduced working memory (Egan et al., 2003; Erickson et al., 2013). Carriers of the ApoE-𝜀4 allele, representing 15–20% of the population, show higher risk of developing AD (Farrer et al., 1997), and physical activity was shown to improve cognition in non-𝜀4 carriers, but not in 𝜀4 carriers, demonstrating the importance to discriminate both populations (Obisesan et al., 2012). Finally, it is important to mention that cognitive training can also have beneficial effects in both young and older adults (Belleville and Bherer, 2012; Lussier et al., 2012).

Collectively, the unbiased approach used here has revealed distinct changes in gene expression, biological processes, and signaling pathways that are triggered in response to voluntary running versus running-independent EE. Our findings can be used to generate data-supported hypotheses concerning novel mediators of the associated anatomical and functional changes within the hippocampus.

## Author Contributions

C-AG, ST, ÉS, SF, and KF: conceived and designed the experiments. C-AG, ST, BG, ÉS, AL, and AA: performed the experiments. C-AG, ST, SF, and KF: analyzed the data. C-AG and KF: wrote the paper. C-AG, ST, BG, ÉS, PD, SF, and KF: revised the paper.

## Conflict of Interest Statement

The authors declare that the research was conducted in the absence of any commercial or financial relationships that could be construed as a potential conflict of interest.

## Footnotes

1 www.qiagen.com/ingenuity
