# Supplementary material for: RNA-Sequencing Reveals Unique Transcriptional Signatures of Running and Running-Independent Environmental Enrichment in the Adult Mouse Dentate Gyrus
Source: Front Mol Neurosci. 2018 Apr 13;11:126. doi: 10.3389/fnmol.2018.00126 (PMC5908890; doi:10.3389/fnmol.2018.00126)
Supplement: Supplementary file 1 [file Table_1.PDF]

Extended Data Figure 4-1. Significantly changed genes in CE

	Experimental group, normalized Log2(Readcount)					
	CE	RUN	H-RUN	LD	L-RUN	Log2FoldChange
Thbs1	6.922349	6.255018	6.3230323	6.2362782	6.24035088	0.686070771
Ttr	8.4542969	8.014302	7.97033266	7.8325179	7.96671097	0.621778965
Atp6v0c	9.1763053	8.629236	8.65206891	8.5764599	8.65036926	0.599845383
Gm14403	8.8703794	9.03817	9.1364119	9.404843	8.93857353	-0.534463644
Gpr101	8.3915469	7.871618	7.56087349	7.8652508	8.10170773	0.526296078
Col6a1	11.769633	11.46307	11.1662918	11.250988	11.7115813	0.518644961
Gm12070	10.779868	10.30629	10.4824931	10.26257	10.5788068	0.517297999
Lgr6	9.0516957	8.946891	8.99703545	8.5464669	8.87011006	0.505228881
Cpne7	12.679462	12.16674	12.0770758	12.195653	12.2721415	0.483809305
Erdr1	12.028099	11.49861	11.3438923	11.545309	11.7542337	0.482790431
Cyp26b1	8.487205	8.856848	8.93353584	8.9661026	8.74955599	-0.478897537
Htr2c	9.1219739	8.506739	8.33497652	8.6511685	8.76818293	0.470805376
Cpne2	10.65913	10.2582	10.1576584	10.198919	10.3871756	0.460210719
Col1a2	8.0185939	7.989086	7.62152779	7.5670323	8.2412155	0.451561586
Cwc22	13.239977	13.31672	13.6864474	13.683578	12.5352682	-0.44360064
Plagl1	10.521799	10.05943	9.89470006	10.080931	10.3213141	0.440868202
Baiap3	9.4678404	9.330554	9.11676133	9.0298975	9.60347372	0.437942944
Col5a1	10.181469	9.739523	9.50071029	9.7438467	9.91862633	0.43762205
Dgkh	10.020243	9.947282	10.2162509	10.452363	9.56689636	-0.432120245
Cd74	5.7228292	5.928969	5.75243068	6.1547477	6.04607055	-0.431918523
Col6a2	9.1839392	8.851277	8.61134885	8.7559977	9.0550662	0.427941494
Glo1	12.090496	12.19129	12.2653273	12.517859	12.0490466	-0.427362934
Grin2a	9.8806262	9.722167	10.0094854	10.296867	9.41690608	-0.416241154
Ppp1cb	13.330911	13.50077	13.5465129	13.735685	13.5283031	-0.404773947
Prkcd	8.7964409	9.001569	9.11395562	9.1988878	8.83084197	-0.402446881
Gm14295	11.429657	11.68037	11.6411664	11.823128	11.6948051	-0.393470824
Eif2c3	8.9007411	8.804076	8.99971726	9.2938816	8.71880591	-0.393140502
Mt1	11.570624	11.49028	11.4380499	11.177852	11.5838767	0.392771902
Hrh3	9.8990536	9.521143	9.51585668	9.5102686	9.64439617	0.388785056
1700020I14Rik	9.3456788	9.351321	9.55973816	9.7338012	9.04968044	-0.388122368
Pcgf3	9.6849757	9.743718	9.81151671	10.073063	9.73738255	-0.388087337
Col1a1	7.9888305	7.788293	7.60336634	7.6014136	7.99784018	0.387416982
Ly6h	12.500025	12.25981	12.1691767	12.115964	12.3354773	0.384060791
Crocc	11.026739	10.89238	10.6870008	10.646675	10.9723868	0.380064019
E130008D07Rik	6.7586519	6.693796	6.76002277	7.1367584	6.70471479	-0.378106575
Gan	7.2879272	7.148425	7.36233145	6.912916	6.79751515	0.375011211
Prmt8	10.744112	10.75117	10.7936674	11.11871	10.6741466	-0.374598708
Scai	11.187096	11.3057	11.3995845	11.561266	11.2923473	-0.374170108
Rasgrp1	13.027884	13.42222	13.6261198	13.397164	13.158232	-0.369279316
Timp2	13.005011	12.53317	12.4843792	12.636088	12.6830323	0.368923493
Ecel1	7.896853	7.630718	7.36928438	7.5279406	7.91677318	0.368912327

Dcn	9.8219679	9.338881	8.93111531	9.4534858	9.73966479	0.368482045
Itpr1	12.421604	12.30909	12.3890718	12.78927	12.184442	-0.367665913
Nrsn2	10.866376	10.63804	10.575177	10.499152	10.7003673	0.367224662
Col23a1	9.5713234	9.272908	9.10594006	9.2044856	9.35320371	0.366837794
Vps13c	11.908497	11.95246	12.0773136	12.272122	11.789591	-0.363625589
Nnat	12.978182	12.85202	12.625796	12.618776	13.1012296	0.359406327
Wnt4	8.8502259	8.630895	8.57627338	8.5017076	8.79474549	0.348518269
Lnpep	8.8004582	8.684128	8.94126423	9.1483558	8.42682867	-0.347897575
Vgll3	8.6586652	8.285899	8.26504306	8.3162063	8.33363454	0.342458879
Crym	12.313314	11.94549	11.8905843	11.971685	12.012378	0.34162899
Pkia	12.968411	13.2185	13.2908487	13.306383	13.2244288	-0.337971767
Sst	11.709331	11.5716	11.4158444	11.372704	11.6750315	0.336626882
Nptxr	9.3764362	8.947424	9.11165608	9.0404104	8.77832555	0.336025788
Klhl11	8.5160236	8.501748	8.58827283	8.8510105	8.29961166	-0.334986997
Zbtb1	9.0646949	9.211658	9.21934078	9.3919233	9.18858598	-0.327228415
Gstp1	11.396018	11.36477	11.4128975	11.069245	11.3991357	0.326773726
Ldb2	8.364081	8.037078	7.95175574	8.0377414	8.09432805	0.326339605
Alg10b	9.3123178	9.237314	9.30365096	9.6330917	9.071627	-0.320773893
Bace2	7.2098359	6.99595	6.87072817	6.8893599	7.02083581	0.320475943
Arl5b	7.6578022	7.73315	7.76640585	7.9768334	7.56415929	-0.31903118
Cadm2	13.323077	13.57067	13.6812916	13.641543	13.524928	-0.318465588
Gpr123	10.710054	10.43121	10.4540755	10.395245	10.4885846	0.314809142
Cd109	7.2508751	7.370516	7.46936275	7.565469	7.19621664	-0.314593864
Necab2	11.080335	10.85115	10.7923212	10.765745	10.8780901	0.314589712
Sulf2	11.266469	10.93144	10.9926598	10.95211	10.9477193	0.314358374
Rprm	10.841064	11.06299	11.038455	10.527412	11.0649474	0.313651552
2410066E13Rik	9.1036089	9.071029	9.15895464	9.4166209	8.91299566	-0.313011956
Jhdm1d	12.071163	12.16362	12.2018872	12.383958	12.2176055	-0.312795909
2310003H01Rik	9.425229	9.416006	9.3429196	9.7378569	9.44904373	-0.312627933
Mif	11.712099	11.5696	11.5554439	11.401136	11.6229308	0.310962403
Mib1	9.9200078	9.811777	10.0107628	10.230397	9.57416742	-0.31038926
Lmbrd2	8.7231525	8.650522	8.8020153	9.0329595	8.44287263	-0.309807058
Ccdc71l	10.606893	10.81101	10.8789102	10.916199	10.8515979	-0.309306615
Fibcd1	10.659663	9.89082	9.83200125	10.351043	9.89522499	0.308619712
Foxo6	9.56941	9.197388	9.22554967	9.2633797	9.28002935	0.306030323
Skil	11.080511	11.07774	11.2155704	11.384166	10.9302563	-0.303655199
Adcy1	15.321523	15.41946	15.5551819	15.624495	15.2508372	-0.30297211
Igsf21	8.7839388	8.546877	8.56952513	8.4809962	8.54433912	0.302942661
Ptgds	11.941482	12.97794	11.5371932	11.640479	13.6104458	0.301003028
Cnot6l	9.0889061	9.152128	9.23119648	9.3891762	8.99181366	-0.30027009
