# Supplementary material for: RNA-Sequencing Reveals Unique Transcriptional Signatures of Running and Running-Independent Environmental Enrichment in the Adult Mouse Dentate Gyrus
Source: Front Mol Neurosci. 2018 Apr 13;11:126. doi: 10.3389/fnmol.2018.00126 (PMC5908890; doi:10.3389/fnmol.2018.00126)
Supplement: Supplementary file 2 [file Table_2.PDF]

Extended Data Figure 4-2. Significantly changed genes in RUN

| | Experimental group, normalized Log2(Readcount) | | | | | |
|---|---|---|---|---|---|---|
| | CE | RUN | H-RUN | LD | L-RUN | Log2FoldChange |
| Ptgds | 11.9414822 | 12.9779408 | 11.5371932 | 11.6404791 | 13.6104458 | 1.337461706 |
| Penk | 10.5185718 | 11.3151772 | 11.2947862 | 10.4604868 | 11.3656483 | 0.854690465 |
| Fmod | 7.68539584 | 8.1829031 | 7.59748785 | 7.58446509 | 8.5975082 | 0.598438013 |
| Nos1 | 9.19258492 | 8.74407151 | 8.86874294 | 9.32254116 | 8.58458017 | -0.578469652 |
| Igf2 | 9.74696701 | 10.2280787 | 9.60346077 | 9.65309257 | 10.7489481 | 0.574986147 |
| Grin2a | 9.88062624 | 9.72216741 | 10.0094854 | 10.2968674 | 9.41690608 | -0.574699978 |
| Htra4 | 8.62509395 | 9.16810414 | 9.19542518 | 8.62401132 | 9.15061246 | 0.544092825 |
| Kcnq3 | 8.85727965 | 8.59921884 | 8.74723548 | 9.13933245 | 8.4509077 | -0.540113609 |
| Rprm | 10.8410639 | 11.0629893 | 11.038455 | 10.5274124 | 11.0649474 | 0.53557688 |
| Parm1 | 8.81094488 | 8.35539522 | 8.4446966 | 8.87501307 | 8.29996834 | -0.519617852 |
| Megf9 | 10.1242169 | 9.91377859 | 10.1026799 | 10.4203484 | 9.66534183 | -0.50656982 |
| Cbl | 9.02374115 | 8.79892013 | 9.05918864 | 9.30466983 | 8.57114885 | -0.505749694 |
| Dgkh | 10.020243 | 9.94728199 | 10.2162509 | 10.4523632 | 9.56689636 | -0.505081219 |
| Igfbp5 | 12.6250156 | 12.1621899 | 12.1132248 | 12.6638244 | 12.1812405 | -0.501634451 |
| Frrs1l | 11.8747906 | 11.61003 | 11.7539126 | 12.1060602 | 11.4448769 | -0.496030221 |
| Eif2c3 | 8.90074105 | 8.80407613 | 8.99971726 | 9.29388155 | 8.71880591 | -0.489805422 |
| Matn2 | 10.0883917 | 9.68608501 | 9.76553411 | 10.1663125 | 9.64121488 | -0.480227475 |
| Itpr1 | 12.4216037 | 12.3090912 | 12.3890718 | 12.7892696 | 12.184442 | -0.480178461 |
| Apod | 10.2188666 | 10.5424897 | 10.0441592 | 10.069032 | 10.8493715 | 0.473457675 |
| Lnpep | 8.80045825 | 8.68412767 | 8.94126423 | 9.14835582 | 8.42682867 | -0.46422815 |
| Eif2c2 | 10.2864465 | 10.059634 | 10.1441487 | 10.5207837 | 9.90047943 | -0.461149726 |
| Fibcd1 | 10.659663 | 9.89081971 | 9.83200125 | 10.3510432 | 9.89522499 | -0.460223533 |
| Trpm3 | 10.8676342 | 10.6603474 | 10.8110026 | 11.1204239 | 10.4724765 | -0.460076587 |
| Robo3 | 10.7194471 | 11.2040824 | 11.1716964 | 10.7447859 | 11.2695991 | 0.459296473 |
| Sorl1 | 11.858558 | 11.6514738 | 11.8245888 | 12.108969 | 11.3433954 | -0.457495274 |
| Tenm1 | 9.21663243 | 8.86597415 | 9.00688795 | 9.32212959 | 8.7629292 | -0.456155438 |
| Ccbe1 | 9.85710057 | 9.59014459 | 9.66974775 | 10.0457353 | 9.54358949 | -0.455590672 |
| Grik3 | 10.4705266 | 9.9627263 | 9.88238656 | 10.4180034 | 10.0457097 | -0.455277068 |
| Igfbp6 | 8.22917694 | 8.58009693 | 8.58451369 | 8.12605239 | 8.68367792 | 0.454044535 |
| Hrk | 11.1289764 | 10.8266844 | 10.8371433 | 11.2723177 | 10.9245761 | -0.445633298 |
| D10Bwg1379e | 10.3547987 | 10.1057253 | 10.3047006 | 10.5487587 | 9.82158269 | -0.443033342 |
| E130008D07Rik | 6.75865186 | 6.69379608 | 6.76002277 | 7.13675844 | 6.70471479 | -0.442962358 |
| Aldh1a2 | 7.30674166 | 7.68200809 | 7.26349435 | 7.24076864 | 8.00070036 | 0.441239456 |
| Grin2b | 8.79722346 | 8.60217459 | 8.79009931 | 9.04337972 | 8.32815727 | -0.44120513 |
| Pcdhac2 | 8.67773816 | 8.37995815 | 8.60202327 | 8.81953091 | 8.27592757 | -0.439572759 |
| Rab11fip4 | 10.5080195 | 10.3099618 | 10.465299 | 10.744436 | 10.1174006 | -0.434474284 |
| Slc13a4 | 7.16863939 | 7.53870273 | 7.08508635 | 7.10662378 | 7.93367749 | 0.432078952 |
| Ppl | 8.67517519 | 9.00944355 | 8.96484192 | 8.57961031 | 9.02176922 | 0.429833239 |
| Col1a2 | 8.01859387 | 7.9890863 | 7.62152779 | 7.56703228 | 8.2412155 | 0.422054023 |
| Bmpr2 | 9.20922425 | 9.01002644 | 9.20705244 | 9.42886846 | 8.76889646 | -0.418842021 |
| Mib1 | 9.92000778 | 9.81177671 | 10.0107628 | 10.230397 | 9.57416742 | -0.418620332 |
| Kif26b | 9.08614888 | 8.93488454 | 9.00928071 | 9.35031291 | 8.80303563 | -0.415428362 |

| | | | | | | |
|---|---|---|---|---|---|---|
| Mgp | 7.41592248 | 7.66840219 | 7.24772673 | 7.25316997 | 7.95845443 | 0.415232215 |
| Fat4 | 11.6970101 | 11.3291672 | 11.3240808 | 11.7433672 | 11.4088816 | -0.414200001 |
| Slc6a13 | 7.20122732 | 7.54075073 | 7.09168414 | 7.12795378 | 7.82186781 | 0.412796951 |
| Nr2c2 | 10.2149399 | 10.0427396 | 10.2591996 | 10.4536329 | 9.88439641 | -0.410893286 |
| Plekha2 | 11.7764956 | 12.2498475 | 12.2953989 | 11.8391995 | 12.1760193 | 0.410647957 |
| Elfn2 | 11.4172822 | 11.0100583 | 11.0653132 | 11.4196548 | 10.9146753 | -0.409596586 |
| Glg1 | 11.0028398 | 10.8298123 | 11.0595339 | 11.2363718 | 10.5067845 | -0.406559461 |
| Spock2 | 13.1103113 | 12.7848413 | 12.8959825 | 13.1878542 | 12.6597989 | -0.403012944 |
| Darc | 9.93472847 | 10.2210355 | 10.1775028 | 9.81943169 | 10.1096243 | 0.401603784 |
| Lgr6 | 9.05169574 | 8.9468912 | 8.99703545 | 8.54646686 | 8.87011006 | 0.400424345 |
| Sh3bgrl3 | 11.7860774 | 11.9784827 | 11.9625349 | 11.5787142 | 12.0101768 | 0.399768499 |
| Alg10b | 9.31231778 | 9.23731369 | 9.30365096 | 9.63309167 | 9.071627 | -0.395777982 |
| Kitl | 10.4541834 | 10.1989132 | 10.1867716 | 10.5946865 | 10.3170766 | -0.395773326 |
| Serinc2 | 7.79717218 | 8.1826361 | 8.19213146 | 7.79274925 | 8.15684879 | 0.389886857 |
| Aebp1 | 7.87955429 | 8.10888055 | 7.69452547 | 7.72112924 | 8.43417179 | 0.387751313 |
| 1500012F01Rik | 9.29469969 | 9.6577612 | 9.63147572 | 9.27015275 | 9.62957111 | 0.387608454 |
| Krt9 | 8.93861404 | 9.25436295 | 9.19510369 | 8.86777134 | 9.17241344 | 0.386591608 |
| Itpkb | 9.41205945 | 9.18735462 | 9.36872669 | 9.57321199 | 9.1704318 | -0.385857368 |
| Nrip1 | 7.92239131 | 7.77300147 | 7.91620025 | 8.15857973 | 7.6028739 | -0.38557826 |
| Setbp1 | 10.4495926 | 10.2679822 | 10.3188744 | 10.6527648 | 10.2619389 | -0.384782588 |
| Uhmk1 | 8.73574795 | 8.6099469 | 8.81064322 | 8.99446289 | 8.38780326 | -0.384515982 |
| Pcdh1 | 11.0612914 | 10.7354858 | 11.0009587 | 11.1190542 | 10.355319 | -0.383568429 |
| 1700020I14Rik | 9.34567884 | 9.35132056 | 9.55973816 | 9.73380121 | 9.04968044 | -0.382480646 |
| Lmbrd2 | 8.72315246 | 8.65052177 | 8.8020153 | 9.03295951 | 8.44287263 | -0.382437746 |
| Synm | 10.4025313 | 10.2567855 | 10.291512 | 10.6383304 | 10.1495119 | -0.381544955 |
| Ptpn4 | 8.82365224 | 8.71347899 | 8.8448245 | 9.09304056 | 8.66834863 | -0.379561574 |
| Cdkl5 | 8.27348851 | 8.18379689 | 8.41162924 | 8.56295405 | 7.82689117 | -0.379157163 |
| Tnfrsf25 | 10.1070955 | 10.4634543 | 10.2654543 | 10.0855068 | 10.646605 | 0.377947538 |
| Stx1b | 11.3403774 | 11.0523904 | 11.2377387 | 11.4275586 | 10.795628 | -0.375168228 |
| Rgs4 | 12.0114453 | 12.4003986 | 12.4463603 | 12.0299143 | 12.4060789 | 0.37048425 |
| D430041D05Rik | 12.6911234 | 12.4557608 | 12.468205 | 12.8253322 | 12.4560397 | -0.369571452 |
| Ccdc171 | 7.45745329 | 7.78246841 | 7.57275204 | 7.41384076 | 7.8629001 | 0.368627651 |
| Prmt8 | 10.7441116 | 10.7511655 | 10.7936674 | 11.1187103 | 10.6741466 | -0.36754486 |
| C1ql2 | 11.859996 | 12.061984 | 12.0990124 | 11.6948471 | 12.0651473 | 0.367136909 |
| Cwc22 | 13.2399773 | 13.3167221 | 13.6864474 | 13.683578 | 12.5352682 | -0.366855821 |
| Gm14403 | 8.87037937 | 9.03817023 | 9.1364119 | 9.40484302 | 8.93857353 | -0.366672788 |
| Nr1d2 | 11.5158144 | 11.3233077 | 11.3443423 | 11.6897983 | 11.3156418 | -0.366490654 |
| Lrrc8b | 9.2505597 | 9.05000145 | 9.26680924 | 9.41441701 | 8.89064628 | -0.364415557 |
| Tmem245 | 8.64403093 | 8.53540119 | 8.69654077 | 8.89906676 | 8.44971716 | -0.363665569 |
| Slc15a2 | 9.91963886 | 9.75348977 | 9.84479256 | 10.116269 | 9.69899277 | -0.36277926 |
| Adamts1 | 9.27817213 | 9.11573617 | 9.16137056 | 9.47692312 | 9.12797064 | -0.361186952 |
| Xrn1 | 8.82682777 | 8.7179881 | 8.94180969 | 9.07835691 | 8.57643298 | -0.36036881 |
| Ppm1l | 9.39348275 | 9.26731401 | 9.47042668 | 9.62726599 | 8.94935222 | -0.35995198 |
| Dio2 | 10.5189126 | 10.4450165 | 10.524905 | 10.8019739 | 10.4086782 | -0.35695742 |
| Megf6 | 7.44563431 | 7.68626156 | 7.60084676 | 7.32956456 | 7.60454008 | 0.356697004 |
| Glt8d2 | 8.66268312 | 8.46432575 | 8.38234017 | 8.81881817 | 8.4252359 | -0.354492414 |

| | | | | | | |
|---|---|---|---|---|---|---|
| Drd1a | 8.17449717 | 8.56101026 | 8.63940785 | 8.20668292 | 8.44640589 | 0.354327344 |
| Tmc6 | 7.79211707 | 8.12138433 | 8.04175236 | 7.76853252 | 8.18227273 | 0.352851809 |
| Vwa3a | 9.76337316 | 9.50161614 | 9.46431759 | 9.85442321 | 9.5670256 | -0.352807066 |
| Dgki | 9.19650531 | 9.1048729 | 9.2988113 | 9.45517917 | 8.94019766 | -0.35030627 |
| Slc22a6 | 6.46630778 | 6.77248573 | 6.41707179 | 6.42229923 | 7.04969815 | 0.350186492 |
| Klhl11 | 8.51602355 | 8.50174823 | 8.58827283 | 8.85101055 | 8.29961166 | -0.349262322 |
| Mtmr9 | 9.86018911 | 9.72591272 | 9.88058375 | 10.0740396 | 9.55688256 | -0.348126847 |
| Homer2 | 9.99914302 | 9.84201428 | 9.93332112 | 10.1890288 | 9.70840237 | -0.347014495 |
| Tmem178b | 7.53692509 | 7.2623647 | 7.34072782 | 7.60902458 | 7.04968625 | -0.346659877 |
| 2410066E13Rik | 9.1036089 | 9.07102918 | 9.15895464 | 9.41662086 | 8.91299566 | -0.345591678 |
| Sgk1 | 10.5992422 | 10.8809697 | 11.0816294 | 10.5356237 | 10.7945364 | 0.345345985 |
| Trip11 | 9.15167749 | 8.98874938 | 9.13822449 | 9.3337401 | 8.82457484 | -0.344990725 |
| Gjb2 | 7.19372526 | 7.43519384 | 7.16689898 | 7.09236492 | 7.76826547 | 0.342828914 |
| Pnmal1 | 10.9919004 | 10.8285661 | 10.9500107 | 11.1709582 | 10.7108301 | -0.342392105 |
| Zbed6 | 10.6369511 | 10.4686536 | 10.5403518 | 10.8106078 | 10.3520788 | -0.341954224 |
| Hlf | 12.1387122 | 11.9322079 | 11.8928466 | 12.2736823 | 12.021205 | -0.341474378 |
| Sema4d | 9.83719701 | 9.57712573 | 9.63884433 | 9.9178265 | 9.51360762 | -0.340700776 |
| Atp2b4 | 12.285273 | 11.8573795 | 11.8452958 | 12.1978527 | 11.9624967 | -0.34047325 |
| Strn | 9.29307271 | 9.14103439 | 9.30771294 | 9.48077386 | 9.02108185 | -0.339739466 |
| Cep85l | 7.37333001 | 7.24635196 | 7.36110364 | 7.5859238 | 7.2151595 | -0.339571837 |
| Tnpo1 | 10.158971 | 10.071955 | 10.176329 | 10.4112266 | 9.91679093 | -0.339271539 |
| Cacna1e | 13.4968159 | 13.430123 | 13.5751849 | 13.7684607 | 13.2616142 | -0.338337624 |
| Pappa | 7.14314488 | 7.49569082 | 7.61415581 | 7.15743364 | 7.27908444 | 0.338257181 |
| Nuak1 | 10.1211215 | 10.0130633 | 9.9598556 | 10.3508027 | 9.99636516 | -0.337739433 |
| Kif17 | 9.86316237 | 10.0886073 | 10.1089328 | 9.75243764 | 10.057612 | 0.336169637 |
| Clmn | 11.6043532 | 11.3599125 | 11.4657863 | 11.6957567 | 11.294787 | -0.335844212 |
| Zhx3 | 9.26091117 | 9.13399285 | 9.29038987 | 9.46978987 | 9.06080377 | -0.335797019 |
| Ubb | 13.6000955 | 13.7824186 | 13.6348312 | 13.4467741 | 13.7765213 | 0.33564454 |
| Iglon5 | 10.3119159 | 10.497299 | 10.554441 | 10.1616942 | 10.5711904 | 0.335604792 |
| Ksr2 | 8.3087639 | 8.12274957 | 8.39304609 | 8.45817893 | 7.88498523 | -0.335429358 |
| Arc | 10.3876277 | 10.0983721 | 10.2782225 | 10.4325401 | 9.97945837 | -0.334167922 |
| Gatad2b | 8.98024938 | 8.78965247 | 9.00677141 | 9.12152207 | 8.64174682 | -0.331869604 |
| Cers6 | 10.0911686 | 10.0165322 | 10.1567511 | 10.3483722 | 9.84710232 | -0.331839929 |
| Fktn | 9.51758308 | 9.43418339 | 9.51502105 | 9.7654314 | 9.32644803 | -0.331248009 |
| Pcgf3 | 9.68497569 | 9.74371762 | 9.81151671 | 10.073063 | 9.73738255 | -0.329345416 |
| Tgfbr1 | 10.3891845 | 10.3337058 | 10.2381644 | 10.6624879 | 10.3904164 | -0.328782097 |
| Csdc2 | 11.6004372 | 11.7811167 | 11.7578108 | 11.453191 | 11.7941274 | 0.327925685 |
| Kcnj16 | 8.94640401 | 8.58438949 | 8.5090532 | 8.91214992 | 8.77190327 | -0.32776043 |
| Mdga1 | 10.435896 | 10.2040488 | 10.2558591 | 10.5309513 | 10.1108871 | -0.326902505 |
| Glo1 | 12.0904963 | 12.1912866 | 12.2653273 | 12.5178592 | 12.0490466 | -0.326572608 |
| Ddn | 15.9201914 | 15.678183 | 15.7832772 | 16.0044032 | 15.542414 | -0.326220245 |
| Pygo1 | 8.5189066 | 8.33786858 | 8.49354249 | 8.66343075 | 8.31199548 | -0.325562163 |
| Nptx2 | 9.50701881 | 9.82123668 | 9.91664056 | 9.49684946 | 9.70008315 | 0.324387216 |
| Gpnmb | 7.80010203 | 8.16922518 | 8.20062115 | 7.84556521 | 7.99460572 | 0.323659965 |
| 2310003H01Rik | 9.42522896 | 9.41600641 | 9.3429196 | 9.73785689 | 9.44904373 | -0.321850482 |
| Tmsb10 | 10.4703643 | 10.6499026 | 10.575588 | 10.3283922 | 10.6990223 | 0.321510459 |

| | | | | | | |
|---|---|---|---|---|---|---|
| Zfp369 | 8.3488381 | 8.23476164 | 8.37643234 | 8.55617349 | 8.00900678 | -0.321411848 |
| Ndst1 | 10.6861718 | 10.5026026 | 10.6736168 | 10.8229818 | 10.2727186 | -0.320379207 |
| Lonrf2 | 11.9294031 | 11.8794457 | 11.938574 | 12.1995736 | 11.747937 | -0.320127923 |
| Vps13c | 11.9084969 | 11.9524604 | 12.0773136 | 12.2721225 | 11.789591 | -0.319662111 |
| Trnp1 | 11.3065425 | 11.4679815 | 11.5493963 | 11.1488296 | 11.4296282 | 0.31915195 |
| Nav3 | 9.61192672 | 9.42802405 | 9.52949191 | 9.74652705 | 9.24801213 | -0.318502999 |
| Cpeb3 | 10.0049122 | 9.87067026 | 10.0273059 | 10.1890017 | 9.81384854 | -0.318331415 |
| Amotl1 | 8.94349695 | 8.75727826 | 8.74987008 | 9.07540104 | 8.91352591 | -0.318122783 |
| Lyst | 12.072787 | 12.0089062 | 12.0922776 | 12.326517 | 11.9223356 | -0.317610806 |
| Kcnip3 | 11.404172 | 11.5463183 | 11.5274414 | 11.2295479 | 11.5188739 | 0.316770424 |
| Cntnap5a | 8.45993615 | 8.41216533 | 8.50672662 | 8.72857355 | 8.25882405 | -0.316408214 |
| Klhdc8b | 9.62191227 | 9.82099998 | 9.77219947 | 9.50608982 | 9.83291525 | 0.314910158 |
| Fam171b | 11.9108314 | 11.7846639 | 11.9038988 | 12.0979915 | 11.5420709 | -0.313327615 |
| Ptprt | 8.30015926 | 7.97124243 | 8.12961716 | 8.28453627 | 7.89809874 | -0.313293844 |
| Dpysl3 | 8.08531374 | 7.91689893 | 8.00577971 | 8.22993266 | 7.85782579 | -0.313033726 |
| Mt1 | 11.5706241 | 11.490283 | 11.4380499 | 11.1778522 | 11.5838767 | 0.312430795 |
| Glp2r | 7.1227972 | 7.24791055 | 7.24547342 | 6.93559919 | 7.12816219 | 0.31231136 |
| Lzts1 | 8.3168794 | 8.24353164 | 8.33869542 | 8.55577919 | 8.13661123 | -0.312247555 |
| Zfp536 | 7.58851731 | 7.38471448 | 7.54598356 | 7.69558519 | 7.27573354 | -0.310870708 |
| Mef2d | 11.043366 | 10.781799 | 10.9598124 | 11.0924415 | 10.6492841 | -0.310642534 |
| Slc9a7 | 7.84017792 | 7.72502521 | 7.90383882 | 8.03525828 | 7.57373087 | -0.310233065 |
| Mef2a | 10.7071975 | 10.5646023 | 10.7280286 | 10.8741449 | 10.4437242 | -0.30954258 |
| Hdac4 | 8.29828539 | 8.0822942 | 8.16357913 | 8.39169274 | 7.90619061 | -0.309398542 |
| Gpr26 | 7.65953844 | 7.45673945 | 7.54781096 | 7.76578891 | 7.33243625 | -0.309049466 |
| Cntnap5b | 9.1607739 | 9.04463366 | 9.20704891 | 9.35356532 | 9.04482785 | -0.30893166 |
| Ecm2 | 8.67250954 | 8.45252774 | 8.42778953 | 8.76139401 | 8.50246211 | -0.308866264 |
| Tnr | 10.8218251 | 10.6731459 | 10.8949959 | 10.9815569 | 10.3256709 | -0.308410937 |
| Ypel4 | 10.1257298 | 10.3435142 | 10.3471169 | 10.0360238 | 10.4203434 | 0.307490393 |
| Flt1 | 9.32930921 | 9.23215328 | 9.31525547 | 9.53956852 | 9.10530312 | -0.307415243 |
| Irs2 | 9.41640193 | 9.12509195 | 9.25639794 | 9.43244809 | 9.01290034 | -0.307356142 |
| Impad1 | 10.5727059 | 10.559361 | 10.6584738 | 10.8666916 | 10.4359572 | -0.307330532 |
| Elovl6 | 10.1739679 | 10.1566288 | 10.2398463 | 10.4639558 | 10.122207 | -0.307327072 |
| Mapk4 | 10.6905434 | 10.9872792 | 10.9704056 | 10.6804548 | 10.9436639 | 0.306824378 |
| Skil | 11.0805111 | 11.077745 | 11.2155704 | 11.3841663 | 10.9302563 | -0.306421332 |
| Prkcb | 13.2239169 | 13.1458867 | 13.3078791 | 13.4508653 | 12.8938153 | -0.304978596 |
| Nebl | 10.0738478 | 10.0511507 | 10.239647 | 10.3560322 | 9.9126857 | -0.304881472 |
| Etv1 | 10.7227528 | 10.3424413 | 10.3297669 | 10.6470704 | 10.3831355 | -0.304629088 |
| Akt3 | 12.3491522 | 12.2590975 | 12.3823338 | 12.5628331 | 12.1024828 | -0.303735539 |
| Ppp1r12b | 9.23734794 | 9.1136974 | 9.28653966 | 9.41716144 | 8.79890148 | -0.303464039 |
| Rap1gap2 | 12.4347488 | 12.1122975 | 12.1716071 | 12.4149548 | 12.073063 | -0.302657248 |
| Qk | 12.7024636 | 12.6843158 | 12.7719943 | 12.9862159 | 12.7107915 | -0.301900138 |
| Grin3a | 9.98888787 | 9.65533852 | 9.62400293 | 9.95659736 | 9.63887838 | -0.301258838 |
| Cdr1 | 9.37228113 | 9.33467842 | 9.44040276 | 9.63575182 | 9.25128121 | -0.301073404 |
| Baiap3 | 9.46784043 | 9.33055421 | 9.11676133 | 9.02989749 | 9.60347372 | 0.300656726 |
