# Supplementary material for: RNA-Sequencing Reveals Unique Transcriptional Signatures of Running and Running-Independent Environmental Enrichment in the Adult Mouse Dentate Gyrus
Source: Front Mol Neurosci. 2018 Apr 13;11:126. doi: 10.3389/fnmol.2018.00126 (PMC5908890; doi:10.3389/fnmol.2018.00126)
Supplement: Supplementary file 3 [file Table_3.PDF]

Extended Data Figure 6-1. Significantly changed genes in L-RUN

	Experimental group, normalized Log2(Readcount)					
	CE	RUN	H-RUN	LD	L-RUN	Log2FoldChange
Ptgds	11.9414822	12.9779408	11.5371932	11.6404791	13.6104458	1.969966664
Cwc22	13.2399773	13.3167221	13.6864474	13.683578	12.5352682	-1.148309763
Igf2	9.74696701	10.2280787	9.60346077	9.65309257	10.7489481	1.095855535
Fmod	7.68539584	8.1829031	7.59748785	7.58446509	8.5975082	1.013043108
Penk	10.5185718	11.3151772	11.2947862	10.4604868	11.3656483	0.905161508
Dgkh	10.020243	9.94728199	10.2162509	10.4523632	9.56689636	-0.885466855
Grin2a	9.88062624	9.72216741	10.0094854	10.2968674	9.41690608	-0.879961316
Slc13a4	7.16863939	7.53870273	7.08508635	7.10662378	7.93367749	0.827053713
Apod	10.2188666	10.5424897	10.0441592	10.069032	10.8493715	0.780339483
Sorl1	11.858558	11.6514738	11.8245888	12.108969	11.3433954	-0.765573667
Pcdh1	11.0612914	10.7354858	11.0009587	11.1190542	10.355319	-0.763735211
Aldh1a2	7.30674166	7.68200809	7.26349435	7.24076864	8.00070036	0.759931722
Megf9	10.1242169	9.91377859	10.1026799	10.4203484	9.66534183	-0.755006575
Nos1	9.19258492	8.74407151	8.86874294	9.32254116	8.58458017	-0.737960989
Cdkl5	8.27348851	8.18379689	8.41162924	8.56295405	7.82689117	-0.736062875
Cbl	9.02374115	8.79892013	9.05918864	9.30466983	8.57114885	-0.73352098
Glg1	11.0028398	10.8298123	11.0595339	11.2363718	10.5067845	-0.729587274
D10Bwg1379e	10.3547987	10.1057253	10.3047006	10.5487587	9.82158269	-0.727176005
Lnpep	8.80045825	8.68412767	8.94126423	9.14835582	8.42682867	-0.721527153
Grin2b	8.79722346	8.60217459	8.79009931	9.04337972	8.32815727	-0.715222453
Aebp1	7.87955429	8.10888055	7.69452547	7.72112924	8.43417179	0.713042544
Mgp	7.41592248	7.66840219	7.24772673	7.25316997	7.95845443	0.705284457
Slc6a13	7.20122732	7.54075073	7.09168414	7.12795378	7.82186781	0.693914032
Kcnq3	8.85727965	8.59921884	8.74723548	9.13933245	8.4509077	-0.688424749
1700020I14Rik	9.34567884	9.35132056	9.55973816	9.73380121	9.04968044	-0.68412077
Ppm1l	9.39348275	9.26731401	9.47042668	9.62726599	8.94935222	-0.677913772
Gjb2	7.19372526	7.43519384	7.16689898	7.09236492	7.76826547	0.675900543
Col1a2	8.01859387	7.9890863	7.62152779	7.56703228	8.2412155	0.674183214
Frrs1l	11.8747906	11.61003	11.7539126	12.1060602	11.4448769	-0.661183309
Bmpr2	9.20922425	9.01002644	9.20705244	9.42886846	8.76889646	-0.659971999
Mib1	9.92000778	9.81177671	10.0107628	10.230397	9.57416742	-0.65622962
Tnr	10.8218251	10.6731459	10.8949959	10.9815569	10.3256709	-0.65588591
Trpm3	10.8676342	10.6603474	10.8110026	11.1204239	10.4724765	-0.647947423
Stx1b	11.3403774	11.0523904	11.2377387	11.4275586	10.795628	-0.631930674
Slc22a6	6.46630778	6.77248573	6.41707179	6.42229923	7.04969815	0.627398914
Rab11fip4	10.5080195	10.3099618	10.465299	10.744436	10.1174006	-0.627035484
Eif2c2	10.2864465	10.059634	10.1441487	10.5207837	9.90047943	-0.620304263
Ppp1r12b	9.23734794	9.1136974	9.28653966	9.41716144	8.79890148	-0.618259961
Uhmk1	8.73574795	8.6099469	8.81064322	8.99446289	8.38780326	-0.606659629
Itpr1	12.4216037	12.3090912	12.3890718	12.7892696	12.184442	-0.604827631
Col3a1	6.89924396	6.96664292	6.69033625	6.70313506	7.29577198	0.592636919
Lmbrd2	8.72315246	8.65052177	8.8020153	9.03295951	8.44287263	-0.590086882
Eif2c3	8.90074105	8.80407613	8.99971726	9.29388155	8.71880591	-0.575075642
Parm1	8.81094488	8.35539522	8.4446966	8.87501307	8.29996834	-0.575044729

Baiap3	9.46784043	9.33055421	9.11676133	9.02989749	9.60347372	0.573576231
Ksr2	8.3087639	8.12274957	8.39304609	8.45817893	7.88498523	-0.573193699
Nr2c2	10.2149399	10.0427396	10.2591996	10.4536329	9.88439641	-0.569236511
Alg10b	9.31231778	9.23731369	9.30365096	9.63309167	9.071627	-0.56146467
Tnfrsf25	10.1070955	10.4634543	10.2654543	10.0855068	10.646605	0.561098243
Tmem178b	7.53692509	7.2623647	7.34072782	7.60902458	7.04968625	-0.559338336
Tenm1	9.21663243	8.86597415	9.00688795	9.32212959	8.7629292	-0.559200388
Igfbp6	8.22917694	8.58009693	8.58451369	8.12605239	8.68367792	0.557625529
Fam135b	9.59170231	9.61241757	9.7147702	9.88884619	9.3313798	-0.557466388
Prkcb	13.2239169	13.1458867	13.3078791	13.4508653	12.8938153	-0.557049967
Fam171b	11.9108314	11.7846639	11.9038988	12.0979915	11.5420709	-0.555920631
Nrip1	7.92239131	7.77300147	7.91620025	8.15857973	7.6028739	-0.555705836
Klhl11	8.51602355	8.50174823	8.58827283	8.85101055	8.29961166	-0.551398886
Ndst1	10.6861718	10.5026026	10.6736168	10.8229818	10.2727186	-0.550263276
Kif26b	9.08614888	8.93488454	9.00928071	9.35031291	8.80303563	-0.547277278
Zfp369	8.3488381	8.23476164	8.37643234	8.55617349	8.00900678	-0.547166711
Gramd1b	10.5373567	10.3748934	10.4677354	10.6503274	10.104511	-0.545816352
Rapgef5	11.8170944	11.7749463	11.8992761	12.0665683	11.522267	-0.544301248
Pcdhac2	8.67773816	8.37995815	8.60202327	8.81953091	8.27592757	-0.543603337
Rprm	10.8410639	11.0629893	11.038455	10.5274124	11.0649474	0.53753504
Cpeb4	9.65839732	9.48094046	9.67656355	9.72761737	9.19485464	-0.53276273
Spock2	13.1103113	12.7848413	12.8959825	13.1878542	12.6597989	-0.528055275
Htra4	8.62509395	9.16810414	9.19542518	8.62401132	9.15061246	0.526601137
Matn2	10.0883917	9.68608501	9.76553411	10.1663125	9.64121488	-0.525097605
Robo3	10.7194471	11.2040824	11.1716964	10.7447859	11.2695991	0.524813209
Lrrc8b	9.2505597	9.05000145	9.26680924	9.41441701	8.89064628	-0.52377073
Mtmr9	9.86018911	9.72591272	9.88058375	10.0740396	9.55688256	-0.517157009
Dgki	9.19650531	9.1048729	9.2988113	9.45517917	8.94019766	-0.514981512
Tnks	10.9140322	10.761552	10.886633	11.0488429	10.5375949	-0.511248036
Nr4a3	8.33414333	8.05895723	8.13661827	8.31534231	7.80605646	-0.509285848
Trip11	9.15167749	8.98874938	9.13822449	9.3337401	8.82457484	-0.509165266
Col6a3	7.36543859	7.62920289	7.30293562	7.36075115	7.86770435	0.506953201
Cacna1e	13.4968159	13.430123	13.5751849	13.7684607	13.2616142	-0.506846444
Elfn2	11.4172822	11.0100583	11.0653132	11.4196548	10.9146753	-0.504979566
Epha6	9.26410126	9.23797334	9.48298987	9.48683259	8.98187227	-0.504960314
2410066E13Rik	9.1036089	9.07102918	9.15895464	9.41662086	8.91299566	-0.503625202
Ccbe1	9.85710057	9.59014459	9.66974775	10.0457353	9.54358949	-0.502145777
Xrn1	8.82682777	8.7179881	8.94180969	9.07835691	8.57643298	-0.501923933
Cers6	10.0911686	10.0165322	10.1567511	10.3483722	9.84710232	-0.501269843
Nav3	9.61192672	9.42802405	9.52949191	9.74652705	9.24801213	-0.498514922
Tnpo1	10.158971	10.071955	10.176329	10.4112266	9.91679093	-0.494435628
Mgat5	7.96381224	7.84734716	7.9916874	8.10664272	7.61225034	-0.494392377
Synm	10.4025313	10.2567855	10.291512	10.6383304	10.1495119	-0.488818503
Kcnj6	10.1947335	10.1073357	10.2760739	10.3682258	9.88173879	-0.486486978
Atp2b3	11.8551661	11.6554033	11.8168934	11.9451577	11.4588679	-0.486289788
Hdac4	8.29828539	8.0822942	8.16357913	8.39169274	7.90619061	-0.485502129
Prss22	6.6482917	6.5654407	6.71513649	6.78180321	6.29693831	-0.484864895
Lars2	11.6651947	12.0404241	12.1987914	11.7783922	12.2622104	0.483818263

Igfbp5	12.6250156	12.1621899	12.1132248	12.6638244	12.1812405	-0.482583848
Vps13c	11.9084969	11.9524604	12.0773136	12.2721225	11.789591	-0.482531516
Nnat	12.9781819	12.8520155	12.625796	12.6187756	13.1012296	0.482454063
Homer2	9.99914302	9.84201428	9.93332112	10.1890288	9.70840237	-0.480626404
Gatad2b	8.98024938	8.78965247	9.00677141	9.12152207	8.64174682	-0.479775253
Klf12	8.99383748	8.92228036	9.03391638	9.19073532	8.7112897	-0.47944562
Abhd2	9.12323593	9.05242871	9.21172386	9.3182963	8.8414373	-0.476858994
Sv2c	8.64025015	8.48761484	8.71217993	8.75161931	8.27690934	-0.474709978
Cntnap5a	8.45993615	8.41216533	8.50672662	8.72857355	8.25882405	-0.4697495
Glo1	12.0904963	12.1912866	12.2653273	12.5178592	12.0490466	-0.468812646
Il1f9	8.36481096	8.44223061	8.61506298	8.5522791	8.08549253	-0.466786574
Gm14403	8.87037937	9.03817023	9.1364119	9.40484302	8.93857353	-0.466269485
A630007B06Rik	8.31590531	8.27042224	8.42063628	8.56831129	8.10450514	-0.463806146
Ddn	15.9201914	15.678183	15.7832772	16.0044032	15.542414	-0.461989281
Slc9a7	7.84017792	7.72502521	7.90383882	8.03525828	7.57373087	-0.461527409
Col6a1	11.7696333	11.4630691	11.1662918	11.2509884	11.7115813	0.460592907
Akt3	12.3491522	12.2590975	12.3823338	12.5628331	12.1024828	-0.460350288
Pnmal1	10.9919004	10.8285661	10.9500107	11.1709582	10.7108301	-0.460128072
Strn	9.29307271	9.14103439	9.30771294	9.48077386	9.02108185	-0.459692012
Zbed6	10.6369511	10.4686536	10.5403518	10.8106078	10.3520788	-0.458528984
Xkr4	8.33459539	8.12473273	8.28310118	8.37509832	7.91914287	-0.455955445
Fibcd1	10.659663	9.89081971	9.83200125	10.3510432	9.89522499	-0.455818256
Hmbox1	8.70360772	8.67780423	8.78823919	8.94611943	8.49167061	-0.454448828
Skil	11.0805111	11.077745	11.2155704	11.3841663	10.9302563	-0.453909991
Arc	10.3876277	10.0983721	10.2782225	10.4325401	9.97945837	-0.453081688
Lonrf2	11.9294031	11.8794457	11.938574	12.1995736	11.747937	-0.451636605
Rims1	10.7751455	10.5745144	10.6426311	10.8366783	10.3866941	-0.449984217
Tmem245	8.64403093	8.53540119	8.69654077	8.89906676	8.44971716	-0.449349601
Ccdc171	7.45745329	7.78246841	7.57275204	7.41384076	7.8629001	0.449059337
Adcy9	10.3433104	10.2396372	10.4083274	10.4111455	9.96385605	-0.447289493
Kcnj3	10.5877057	10.5930879	10.6924858	10.7664029	10.3204659	-0.445937005
Pcdhgc3	9.93677196	9.64765681	9.77925543	9.85174224	9.4064553	-0.445286938
Prmt8	10.7441116	10.7511655	10.7936674	11.1187103	10.6741466	-0.444563712
Nebl	10.0738478	10.0511507	10.239647	10.3560322	9.9126857	-0.443346452
Mef2d	11.043366	10.781799	10.9598124	11.0924415	10.6492841	-0.443157423
Trpc5	7.84631966	7.85206549	7.90939855	8.05731706	7.61487454	-0.442442516
Ppl	8.67517519	9.00944355	8.96484192	8.57961031	9.02176922	0.442158916
BC005561	8.7156652	8.64701093	8.78247597	8.85817469	8.4165542	-0.441620487
Lrp1b	11.1277888	11.042819	11.1282018	11.3286288	10.8877152	-0.440913596
Diap1	10.6202506	10.5085891	10.614513	10.7402589	10.2994192	-0.440839701
Slit3	10.8303916	10.5319049	10.632825	10.8281265	10.3885331	-0.439593413
Fktn	9.51758308	9.43418339	9.51502105	9.7654314	9.32644803	-0.438983362
Flt1	9.32930921	9.23215328	9.31525547	9.53956852	9.10530312	-0.434265404
Gpr26	7.65953844	7.45673945	7.54781096	7.76578891	7.33243625	-0.433352667
E130008D07Rik	6.75865186	6.69379608	6.76002277	7.13675844	6.70471479	-0.432043644
Sh3bgrl3	11.7860774	11.9784827	11.9625349	11.5787142	12.0101768	0.431462648
Impad1	10.5727059	10.559361	10.6584738	10.8666916	10.4359572	-0.430734327
Mef2a	10.7071975	10.5646023	10.7280286	10.8741449	10.4437242	-0.430420719

Slc13a3	8.22494903	8.34990701	8.20333705	8.17114298	8.60139566	0.430252681
Pcolce	6.65904533	6.76023861	6.55301209	6.46228232	6.8921469	0.429864578
Arap2	10.2578782	10.260799	10.3570811	10.4862205	10.0585969	-0.427623645
Vtn	10.3578158	10.51455	10.3103671	10.2921784	10.7197226	0.427544128
Nt5dc3	12.6621475	12.5866401	12.7281481	12.8452419	12.4180075	-0.427234432
Ptpn4	8.82365224	8.71347899	8.8448245	9.09304056	8.66834863	-0.424691926
Pou3f1	7.87543858	7.75785729	8.04928127	7.94902677	7.52865232	-0.420374444
AI506816	8.69720975	8.87857426	8.6756231	8.63598421	9.05628195	0.420297743
Mdga1	10.435896	10.2040488	10.2558591	10.5309513	10.1108871	-0.420064126
Zfp536	7.58851731	7.38471448	7.54598356	7.69558519	7.27573354	-0.419851644
Irs2	9.41640193	9.12509195	9.25639794	9.43244809	9.01290034	-0.419547749
Lzts1	8.3168794	8.24353164	8.33869542	8.55577919	8.13661123	-0.419167964
Slc15a2	9.91963886	9.75348977	9.84479256	10.116269	9.69899277	-0.417276255
Fam217b	8.82777648	8.66180043	8.74902305	8.9199044	8.5027557	-0.417148699
Zkscan16	9.25777494	9.19065023	9.29539033	9.41617813	9.00197086	-0.414207274
Tmc6	7.79211707	8.12138433	8.04175236	7.76853252	8.18227273	0.41374021
Hivep3	9.26814521	9.00397626	9.11628662	9.27838218	8.86468938	-0.413692803
Arhgap5	11.2474884	11.1583616	11.2977709	11.43373	11.0206775	-0.413052456
Arl5b	7.65780222	7.73315032	7.76640585	7.9768334	7.56415929	-0.412674109
Prkar2a	10.0280104	9.93040512	9.90966899	10.201319	9.79142861	-0.409890374
Iglon5	10.3119159	10.497299	10.554441	10.1616942	10.5711904	0.409496208
Kctd16	7.26853918	7.21647808	7.30433784	7.45142038	7.04192569	-0.409494691
Ints10	10.8887342	10.8483864	10.8450588	11.1314237	10.7221717	-0.409252014
Zhx3	9.26091117	9.13399285	9.29038987	9.46978987	9.06080377	-0.408986093
Cdh7	7.29001203	7.4931009	7.5086326	7.2645719	7.67346686	0.408894961
Slc8a1	12.1169604	11.8231714	11.9524851	12.0572177	11.6487255	-0.40849222
Adarb2	10.299784	10.2336263	10.3650344	10.4285834	10.0203694	-0.408214023
Prox1	11.8608246	11.7594016	11.9277824	12.014139	11.6066479	-0.407491068
Mt1	11.5706241	11.490283	11.4380499	11.1778522	11.5838767	0.406024555
Nupl1	10.0572209	9.99468233	10.0871624	10.2551864	9.84930709	-0.405879335
Fgd4	8.86709617	8.82876751	8.91815318	9.00409234	8.59835793	-0.405734412
Serping1	7.60073168	7.76069868	7.63975797	7.55985452	7.96532477	0.405470252
Ddi2	7.90756176	7.84235667	7.9715252	8.10067264	7.69534032	-0.405332316
Synj2	9.50568337	9.27226912	9.33124778	9.50670498	9.10152925	-0.405175727
Sema4d	9.83719701	9.57712573	9.63884433	9.9178265	9.51360762	-0.404218884
Lyst	12.072787	12.0089062	12.0922776	12.326517	11.9223356	-0.404181372
Helz	8.51222617	8.37126219	8.53554038	8.60774634	8.20405594	-0.403690403
Ypel3	12.2893181	12.4514707	12.4238596	12.1605865	12.5640939	0.403507363
Pex5l	10.5886613	10.4991824	10.5608491	10.6980209	10.2948014	-0.403219466
Itpkb	9.41205945	9.18735462	9.36872669	9.57321199	9.1704318	-0.402780188
Gpam	9.52936626	9.42900405	9.53234093	9.65518761	9.25278603	-0.402401581
Nr3c2	11.8082202	11.7304637	11.8492698	11.9498663	11.5486747	-0.401191565
Clmn	11.6043532	11.3599125	11.4657863	11.6957567	11.294787	-0.400969719
Cog5	9.26384785	9.24973064	9.3492973	9.52602527	9.12576706	-0.400258212
Garem	9.82060393	9.76402841	9.88263912	9.98226697	9.58296187	-0.399305099
Cnot6l	9.08890615	9.15212798	9.23119648	9.38917624	8.99181366	-0.397362581
Aff2	7.87105948	7.78706538	7.88975242	8.00785817	7.61098169	-0.396876487
Col1a1	7.98883054	7.78829295	7.60336634	7.60141356	7.99784018	0.396426621

Rn45s	14.0768613	14.3771818	14.5809235	14.2639629	14.660353	0.396390024
Slc36a4	8.33866856	8.25651804	8.40262878	8.55245863	8.15665205	-0.395806587
Ppp1r12a	10.7258106	10.6042344	10.7009356	10.8027084	10.4082594	-0.39444903
S100pbp	8.1260051	8.08450533	8.15814226	8.36280948	7.96853936	-0.39427012
Glt8d2	8.66268312	8.46432575	8.38234017	8.81881817	8.4252359	-0.393582269
Dio2	10.5189126	10.4450165	10.524905	10.8019739	10.4086782	-0.393295692
Gm19757	7.58165995	7.60835077	7.78109776	7.79293038	7.40111435	-0.391816032
Dlgap2	10.3278276	10.2196642	10.3838744	10.443477	10.0519075	-0.391569483
Itih2	5.9755624	6.08596618	5.9607872	5.92989169	6.32101091	0.39111922
4933409K07Rik	9.39323778	9.15595185	9.33494405	9.1379067	9.52885127	0.390944573
Setbp1	10.4495926	10.2679822	10.3188744	10.6527648	10.2619389	-0.390825899
Ecel1	7.89685297	7.63071796	7.36928438	7.52794064	7.91677318	0.388832538
Dnajb14	7.79013862	7.70650806	7.87657709	7.96261529	7.57417298	-0.388442313
Igfbp2	9.26808635	9.29486611	9.09387223	9.13153003	9.51903155	0.387501516
Itgb8	8.82228282	8.74206076	8.8830724	8.83980006	8.45264292	-0.387157134
Ccdc88c	8.51041042	8.19202846	8.26636354	8.46085687	8.0737254	-0.387131468
Ptprt	8.30015926	7.97124243	8.12961716	8.28453627	7.89809874	-0.386437531
Ncam2	10.4593033	10.3937751	10.5250771	10.5397556	10.1551211	-0.384634507
Cdr1	9.37228113	9.33467842	9.44040276	9.63575182	9.25128121	-0.384470606
Ypel4	10.1257298	10.3435142	10.3471169	10.0360238	10.4203434	0.384319516
Tnik	12.3397672	12.2397216	12.3281627	12.4478339	12.0638861	-0.383947757
Cacna1c	11.4909123	11.3511415	11.4200951	11.6416596	11.2590045	-0.382655094
Eif4ebp2	8.243934	8.12040485	8.20625145	8.37577529	7.99354202	-0.382233271
Zbtb38	9.53504116	9.38376479	9.48772533	9.60253832	9.22065592	-0.381882397
Caln1	9.20374684	8.97900223	9.11930218	9.2276603	8.84629889	-0.381361417
Bgn	8.39973411	8.41306245	8.27943729	8.21248712	8.59269545	0.380208324
Scand1	9.13609133	9.11955772	9.09655333	8.87807619	9.25780822	0.379732028
Wapal	10.2741878	10.3022633	10.3844615	10.5243258	10.1455723	-0.378753491
Kcnk9	7.22886056	7.17291387	7.25056293	7.34190495	6.96341452	-0.378490431
Pds5a	10.0530885	10.0397931	10.0907665	10.3202602	9.9423444	-0.377915833
Sgtb	10.5090134	10.4987753	10.6161637	10.7355727	10.3582988	-0.377273897
Rgs4	12.0114453	12.4003986	12.4463603	12.0299143	12.4060789	0.376164562
Anxa2	7.40576922	7.39625607	7.23990626	7.2529482	7.62826704	0.375318838
Cpeb3	10.0049122	9.87067026	10.0273059	10.1890017	9.81384854	-0.375153127
Bicd1	8.56975121	8.48318773	8.57175098	8.76924052	8.39480517	-0.374435349
Nr1d2	11.5158144	11.3233077	11.3443423	11.6897983	11.3156418	-0.374156527
Adcy1	15.321523	15.4194646	15.5551819	15.6244951	15.2508372	-0.373657924
Paqr8	11.2151796	11.0455656	11.0780407	11.3409142	10.9678364	-0.373077799
Rgs7bp	12.9741408	12.9744911	13.0762608	13.2291281	12.8563785	-0.37274961
Sv2b	12.4679362	12.2601409	12.4297717	12.4290333	12.0562949	-0.372738458
Grik3	10.4705266	9.9627263	9.88238656	10.4180034	10.0457097	-0.372293681
Dpysl3	8.08531374	7.91689893	8.00577971	8.22993266	7.85782579	-0.372106863
Nos1ap	10.0204186	9.70303475	9.82912753	9.95488099	9.58311942	-0.371761572
Cep85l	7.37333001	7.24635196	7.36110364	7.5859238	7.2151595	-0.370764303
Tmsb10	10.4703643	10.6499026	10.575588	10.3283922	10.6990223	0.370630098
C1ql2	11.859996	12.061984	12.0990124	11.6948471	12.0651473	0.370300255
Cnksr2	12.5197486	12.4283605	12.605909	12.6083092	12.2384979	-0.369811318
D430041D05Rik	12.6911234	12.4557608	12.468205	12.8253322	12.4560397	-0.369292596

Cd109	7.25087509	7.37051571	7.46936275	7.56546895	7.19621664	-0.369252312
Tcp11l1	7.39226905	7.25339452	7.33759019	7.42915702	7.0609488	-0.368208219
Prkcd	8.79644095	9.00156935	9.11395562	9.19888783	8.83084197	-0.36804586
Bmp6	6.56477919	6.78910228	6.65457173	6.54738177	6.91539209	0.36801032
Prepl	12.603916	12.5248122	12.6960227	12.6991512	12.3314458	-0.367705433
Klhl3	9.04812161	8.83971758	8.9639898	9.06283682	8.69545237	-0.36738445
Atp8a2	7.93011008	7.76848618	7.88080027	8.01914355	7.65220187	-0.366941677
D830031N03Rik	6.88662717	6.79698737	6.83685603	7.07732387	6.71052614	-0.366797728
Cpne8	9.54684022	9.61407881	9.53062245	9.47904548	9.8455387	0.366493222
Lyz2	6.49952869	6.64430687	6.50710275	6.51455261	6.88021885	0.365666231
Kcnma1	10.7709953	10.6899968	10.8361762	10.9229264	10.5575054	-0.365421051
Rbp1	7.02652879	7.03133337	6.88569875	6.880317	7.24512475	0.36480775
Serinc2	7.79717218	8.1826361	8.19213146	7.79274925	8.15684879	0.36409954
Ptch1	8.91682687	8.73786772	8.86638648	9.01997004	8.65645215	-0.36351789
Pdpr	8.05814589	8.00522528	8.09195074	8.20396556	7.84051962	-0.363445943
Slc22a8	8.52274709	8.73293567	8.62368321	8.5305101	8.89350885	0.362998749
Zfp382	7.19042523	7.19440202	7.22871331	7.41530135	7.05269067	-0.362610684
Rasl10a	11.2487418	11.5405204	11.4645383	11.248278	11.609192	0.360913999
Chrm3	8.49748161	8.33304735	8.57412985	8.52429811	8.16419473	-0.360103382
Ube3a	10.0932304	10.1057762	10.1323515	10.3599538	10.0002425	-0.359711333
Plk5	11.2511486	11.3659409	11.2507176	11.1112855	11.4709068	0.359621279
1500012F01Rik	9.29469969	9.6577612	9.63147572	9.27015275	9.62957111	0.359418365
Nfix	11.4192052	11.1289748	11.3125664	11.3204634	10.9614178	-0.359045567
Slc6a20a	6.67541111	6.79440026	6.62005988	6.63650676	6.9950882	0.358581434
C1qtnf4	11.5695067	11.5842222	11.577896	11.3084583	11.6667135	0.358255201
St8sia1	9.70769678	9.67854851	9.80617193	9.95135044	9.59352793	-0.357822505
Slc1a2	15.8554878	15.87711	16.0303877	16.0573558	15.6998131	-0.357542752
Dkk3	12.1272848	12.1543984	12.3119805	12.2569162	11.9007238	-0.356192333
Tbck	8.30977903	8.20456857	8.38618111	8.46417349	8.10932371	-0.354849772
Nuak1	10.1211215	10.0130633	9.9598556	10.3508027	9.99636516	-0.354437533
Zfp398	8.36033316	8.29217315	8.34985119	8.48132313	8.1276906	-0.353632524
Rab40b	11.4771613	11.7271236	11.758537	11.451827	11.805146	0.353318996
Ttll7	11.6775239	11.7017644	11.8196177	11.8765954	11.5245004	-0.352095045
Wasf3	10.2599472	10.1478978	10.2291669	10.3473314	9.99563448	-0.351696877
Isyna1	9.45483118	9.40909025	9.28318944	9.2131998	9.56475681	0.35155701
Pygo1	8.5189066	8.33786858	8.49354249	8.66343075	8.31199548	-0.351435268
Tenm3	10.6541291	10.4105115	10.4697889	10.6223447	10.2717301	-0.350614563
Mta3	10.0605347	9.89637207	9.9973118	10.098251	9.74766389	-0.350587153
Add2	13.7222387	13.7435019	13.8002992	13.9954512	13.6453398	-0.350111399
Acot13	10.5460774	10.6231279	10.5141472	10.3842043	10.7335002	0.349295852
Adamts1	9.27817213	9.11573617	9.16137056	9.47692312	9.12797064	-0.34895248
Syt10	7.46647236	7.43961697	7.55480767	7.58759867	7.23921721	-0.348381461
Rpl36	11.2955112	11.315097	11.2269025	11.1149843	11.4629181	0.347933766
Hrk	11.1289764	10.8266844	10.8371433	11.2723177	10.9245761	-0.347741668
Foxo3	8.60697497	8.4485372	8.61079978	8.63150334	8.28501621	-0.346487128
Appbp2	9.62630966	9.48971879	9.61736522	9.74559117	9.39928326	-0.346307905
Nr4a1	8.95133548	8.76261797	8.79774112	8.88558455	8.53928361	-0.34630094
Hexdc	9.13225666	8.9486754	8.98603877	9.13602954	8.78985748	-0.346172057

Slc47a1	5.17052898	5.29927464	5.15033504	5.12515223	5.47093414	0.345781909
Slc24a2	13.9096092	13.8998036	14.0690858	14.0848853	13.7401844	-0.344700903
Kif1b	14.1197199	14.0555059	14.1578738	14.2731988	13.9287166	-0.344482209
Slit1	12.7372226	12.4976933	12.6544873	12.7652843	12.4216808	-0.343603506
Grp	9.28632184	9.44618834	9.36861687	9.20694818	9.55047354	0.343525357
Rap1gap2	12.4347488	12.1122975	12.1716071	12.4149548	12.073063	-0.341891732
Elovl6	10.1739679	10.1566288	10.2398463	10.4639558	10.122207	-0.341748842
Camk1d	12.4770644	12.3903232	12.5028442	12.6114057	12.2697101	-0.341695636
Csdc2	11.6004372	11.7811167	11.7578108	11.453191	11.7941274	0.340936421
Romo1	9.46659965	9.48771801	9.41807036	9.26998918	9.61084756	0.340858378
Map3k13	8.92006845	8.90331023	9.06354548	9.16970788	8.82966529	-0.340042591
Vwa5b2	10.6031719	10.5944332	10.5731801	10.3822254	10.7222183	0.339992882
Neurod6	11.4553436	11.4210233	11.6428096	11.4716061	11.1317017	-0.339904424
Sash1	10.5902577	10.5968206	10.6739945	10.744823	10.4053776	-0.33944548
Mkln1	10.170737	10.1369379	10.2429121	10.3773886	10.0384125	-0.338976154
Dnajc27	11.3832874	11.258591	11.359479	11.4957862	11.1582852	-0.337501033
Gjb6	10.097347	10.3466859	10.2538727	10.1255582	10.4629553	0.337397086
Plekha2	11.7764956	12.2498475	12.2953989	11.8391995	12.1760193	0.336819784
Fat3	11.4893868	11.4081502	11.4513487	11.6721535	11.3359185	-0.336234999
Hist1h2bc	9.76506001	10.0672585	10.0489105	9.80249163	10.1386818	0.336190157
Pcgf3	9.68497569	9.74371762	9.81151671	10.073063	9.73738255	-0.335680478
Dcc	10.5178394	10.398131	10.3906064	10.6682236	10.3326354	-0.335588201
Pcdhga11	8.30553869	8.18595572	8.31092743	8.39329629	8.0583155	-0.334980789
Bbc3	8.33459894	8.41319606	8.36752602	8.17712036	8.51177324	0.334652877
Fat4	11.6970101	11.3291672	11.3240808	11.7433672	11.4088816	-0.334485522
Egr3	9.33519937	9.14865351	9.26357046	9.30539549	8.97104097	-0.334354511
Wfs1	10.5126809	10.4138142	10.6207837	10.4431175	10.1092568	-0.333860667
Mdm4	10.2494405	10.2066302	10.2686059	10.4626214	10.129078	-0.333543461
Sh2d5	11.2952488	11.4532094	11.4606801	11.1997459	11.5326971	0.332951145
Colec12	7.5858217	7.67950268	7.51977835	7.59976423	7.93212977	0.33236554
Naa25	9.82890707	9.77579217	9.89919152	9.96932971	9.63716157	-0.332168138
Cfh	8.67804002	8.86085669	8.630789	8.73336669	9.06545196	0.332085268
Mpped1	10.5789668	10.2918301	10.3901134	10.5116981	10.1798829	-0.331815189
Resp18	10.2836223	10.1737683	9.98020978	9.99637087	10.3273724	0.331001502
Phf20	11.5457113	11.5086015	11.5952979	11.7594282	11.4287705	-0.330657663
Gstp1	11.3960183	11.3647691	11.4128975	11.0692446	11.3991357	0.329891054
Ubb	13.6000955	13.7824186	13.6348312	13.4467741	13.7765213	0.329747179
Nbea	13.6693711	13.6375792	13.7401473	13.8536607	13.5239366	-0.329724104
Cp	7.73630021	7.95525655	7.65056411	7.834598	8.16429847	0.329700468
Gabbr2	12.6267825	12.3108985	12.4441807	12.4671022	12.1379022	-0.329200046
Copg2	10.0258644	9.99153343	10.0985003	10.1571185	9.8282832	-0.328835322
Xylt1	6.87539046	6.69576867	6.76118716	6.91366222	6.58511142	-0.328550799
Rbm33	12.0054663	11.9606035	11.9997878	12.221061	11.8929069	-0.328154133
Col9a2	6.83831779	6.92058584	6.72083873	6.73938115	7.06733892	0.327957769
Klhl34	10.0757252	10.0024944	10.1562186	10.1709642	9.84380543	-0.327158756
Klhdc8b	9.62191227	9.82099998	9.77219947	9.50608982	9.83291525	0.326825427
Soga3	10.7482031	10.6980889	10.8095496	10.8404711	10.514587	-0.325884129
Crocc	11.0267387	10.8923843	10.6870008	10.6466747	10.9723868	0.325712071

Sp140	8.1111404	8.25546249	8.34637898	8.3553002	8.02960811	-0.325692088
Lrrc7	10.6924331	10.6005424	10.6584355	10.7826412	10.4571516	-0.325489633
Mtr	8.04271395	7.83639344	7.90557572	8.12420247	7.79901465	-0.325187827
Pnrc1	10.2929535	10.4341134	10.38149	10.2213306	10.5458724	0.324541784
Ccnt1	8.43167832	8.30078451	8.40605128	8.51276934	8.18844462	-0.324324717
Foxn3	7.26540754	7.18381185	7.28073698	7.38488623	7.06070665	-0.324179574
Tyro3	10.0977335	9.86905031	9.9573938	10.1233982	9.79942968	-0.323968491
Btbd7	8.35051673	8.2392194	8.32006994	8.40325963	8.07931113	-0.323948499
Lgr6	9.05169574	8.9468912	8.99703545	8.54646686	8.87011006	0.3236432
Selm	10.9072586	10.9606372	10.935014	10.6647492	10.9876014	0.322852208
Raver2	9.85558099	9.84244044	9.91250791	10.0272221	9.7044935	-0.322728646
Gpr37	10.116608	10.0542748	10.0560583	10.1775817	9.8551833	-0.322398399
Cacna1b	11.3739274	11.1787716	11.2779411	11.3541184	11.031793	-0.322325416
Tbcd	10.002387	9.86531871	9.92724593	10.0501281	9.72793215	-0.322196001
Bex1	9.97408444	9.96162333	9.85060878	9.78949053	10.1115721	0.322081525
Alkbh8	8.00046015	7.8627704	7.9908761	8.10793291	7.78606609	-0.321866825
Fam13b	10.459061	10.4667426	10.4761127	10.6738155	10.3520287	-0.321786767
Arhgap20	11.6921663	11.6667215	11.7347218	11.8964239	11.5756076	-0.320816269
Cox7b	12.5205421	12.5003628	12.4242693	12.2503906	12.5711751	0.32078452
Spred1	10.7156908	10.6795898	10.7572416	10.8979678	10.5773293	-0.320638578
Ylpm1	11.702874	11.6035065	11.6856931	11.8224158	11.5018421	-0.320573732
Atf7ip	10.4083825	10.2458344	10.3265306	10.4900643	10.1703154	-0.319748936
Jmjd4	9.87239707	9.79142922	9.86170094	9.98825197	9.66867068	-0.319581293
Tmem200a	8.06356305	7.84881893	7.95596574	8.07392597	7.75443966	-0.319486315
Wwc2	9.45141673	9.30983328	9.45586865	9.50890933	9.18943145	-0.319477882
Pcdh19	11.9593154	11.8516685	11.9351396	12.132432	11.8136989	-0.318733057
A830018L16Rik	11.1902005	10.9647592	11.0864945	11.2524951	10.9341978	-0.318297285
Cnot6	9.75114795	9.81094006	9.91084905	10.0389607	9.72092269	-0.318037999
Grin3a	9.98888787	9.65533852	9.62400293	9.95659736	9.63887838	-0.31771898
Chrna7	8.77397364	8.55485642	8.73692954	8.81613385	8.49850969	-0.317624162
3110047P20Rik	10.0634028	10.0116083	10.0227974	10.1931102	9.87556619	-0.317543996
Rgmb	9.93430709	9.69721437	9.74611627	9.93613536	9.61920958	-0.316925776
Npy	10.1269339	10.2729083	10.1173153	10.1189625	10.4357885	0.31682607
Gm12070	10.7798676	10.3062905	10.4824931	10.2625696	10.5788068	0.316237214
Plat	10.5160416	10.629864	10.5752706	10.3669928	10.6831486	0.316155873
Vim	9.54568372	9.60763931	9.46727744	9.45455597	9.7705151	0.315959135
Lepr	7.04375845	7.29957521	7.1576509	7.10088903	7.41651049	0.315621456
Prickle2	12.6976995	12.7625928	12.8552286	12.9321863	12.6168215	-0.31536481
Pcdhgc5	10.869642	10.6144078	10.8206772	10.6881459	10.3740601	-0.314085793
Kat6a	11.2105664	11.0698273	11.1565605	11.2736505	10.9597014	-0.313949025
Scarb2	9.98140172	9.88685482	9.89263218	10.0469479	9.73324236	-0.313705549
Psd3	13.4643442	13.3559984	13.4728847	13.5687406	13.2553787	-0.313361896
C230091D08Rik	9.81419209	9.83989136	9.91342803	10.0952878	9.78193071	-0.313357058
Med12l	10.6326593	10.4806416	10.6209437	10.7677805	10.4552746	-0.312505875
Ntrk2	13.0074605	12.7967237	12.8608845	13.0363591	12.7240796	-0.312279484
Mob1a	7.17248469	7.11417496	7.20101168	7.33590522	7.02365849	-0.312246735
Clec3b	5.54922199	5.8088498	5.61137112	5.60454154	5.91675936	0.312217823
Prex2	10.5841141	10.6920278	10.8113377	10.8825861	10.570815	-0.31177108

E330009J07Rik	7.02643819	6.96281746	7.09075465	7.16168454	6.8499777	-0.311706841
Pde1a	11.9351237	11.5994762	11.6860929	11.8589724	11.5474791	-0.311493317
Kcng3	5.73299265	5.57217852	5.61532584	5.86824665	5.55745524	-0.31079141
Dyrk2	8.13722714	8.03582052	8.09919422	8.22514749	7.91445527	-0.310692217
Cox5b	12.4048794	12.3700622	12.2937171	12.1293082	12.4399717	0.31066349
Kif3b	11.0307042	11.0378459	11.1230528	11.2503298	10.9397258	-0.31060405
Hinfp	8.07399731	8.01982285	8.0754815	8.2090914	7.89853669	-0.310554716
Plcxd2	9.53789557	9.5297005	9.63247281	9.73303324	9.42248645	-0.310546794
Syt14	6.51306328	6.48517128	6.51283687	6.70680402	6.39673042	-0.310073597
Kif5c	14.3836302	14.3542512	14.4612374	14.5704076	14.2615328	-0.308874789
Cntnap5b	9.1607739	9.04463366	9.20704891	9.35356532	9.04482785	-0.308737468
Camk2a	16.7750889	16.6749667	16.8092747	16.8069444	16.4985726	-0.308371808
Scrn1	10.2438426	10.0906041	10.1992511	10.3164983	10.0082445	-0.308253789
Gpx3	7.00669393	7.03035055	6.83029358	6.88104855	7.18907819	0.308029638
Paqr9	9.66240486	9.63570166	9.70034708	9.81958434	9.51292683	-0.306657505
Nhlrc2	7.80614643	7.69491815	7.73945455	7.90344491	7.59701845	-0.306426459
Erc1	11.1517604	10.9901606	11.0828469	11.2500382	10.9437105	-0.306327715
Shisa6	12.1974855	12.0838466	12.1843562	12.2942794	11.9880398	-0.306239527
Nrcam	13.357955	13.2780388	13.3770989	13.4274552	13.1215229	-0.305932273
Nrip3	13.5574748	13.4867149	13.501883	13.7405999	13.4351543	-0.305445566
Kif17	9.86316237	10.0886073	10.1089328	9.75243764	10.057612	0.305174369
Krt9	8.93861404	9.25436295	9.19510369	8.86777134	9.17241344	0.304642099
Rcor1	8.75033786	8.66250595	8.69965046	8.86910665	8.56487007	-0.304236572
Vps33a	11.4004696	11.3433146	11.377515	11.5409882	11.2372578	-0.303730447
Bhlhe40	10.9581102	10.8380831	10.7468253	11.1050407	10.8013987	-0.303641958
Ucp2	9.5307698	9.64402425	9.57166007	9.39999868	9.70343062	0.303431949
Nmt1	11.5487024	11.4932116	11.6142066	11.6919345	11.3886413	-0.303293121
Spink8	6.68547348	6.62499666	6.66885922	6.80827588	6.50508533	-0.30319055
Bmp1	12.4865099	12.6849533	12.6444836	12.3902911	12.6934806	0.303189566
Spint1	7.31975295	7.4055556	7.33198748	7.13804005	7.44071019	0.302670136
Rpl37	12.1388065	12.1713394	12.0981354	11.9963166	12.2988749	0.302558344
Prrc2b	14.0399462	13.9492715	14.032161	14.1531753	13.850816	-0.302359315
Sst	11.7093312	11.5715979	11.4158444	11.3727043	11.6750315	0.302327169
Hspa1b	8.79384448	8.93197907	8.9355579	8.69540124	8.99771208	0.302310839
Kif5a	14.899269	14.665377	14.726382	14.8780568	14.575759	-0.302297764
Hectd1	12.1151627	12.0310563	12.1160461	12.2524485	11.9502317	-0.302216789
3110035E14Rik	11.3914741	11.3139017	11.4336812	11.4837617	11.1822438	-0.301517824
Larp4b	10.4415595	10.3560663	10.4632528	10.5973233	10.2962281	-0.301095233
Slc1a3	13.8854506	13.7679272	13.8077689	14.0147008	13.7137585	-0.300942242
Nid1	7.39290532	7.46860397	7.36599446	7.32577699	7.62666112	0.30088413
Dgkz	13.6316783	13.5160163	13.6134771	13.6828121	13.3820799	-0.300732226
Nrgn	15.2276264	15.3376665	15.3905698	15.0676206	15.3681279	0.300507274
Slc24a4	9.09077868	8.89845805	9.06022228	9.12888118	8.82868548	-0.3001957
Slc29a1	9.16504928	9.25217135	9.2287973	9.0143292	9.31445575	0.300126547
