# Supplementary material for: RNA-Sequencing Reveals Unique Transcriptional Signatures of Running and Running-Independent Environmental Enrichment in the Adult Mouse Dentate Gyrus
Source: Front Mol Neurosci. 2018 Apr 13;11:126. doi: 10.3389/fnmol.2018.00126 (PMC5908890; doi:10.3389/fnmol.2018.00126)
Supplement: Supplementary file 4 [file Table_4.PDF]

Extended Data Figure 6-2. Significantly changed genes in H-RUN

	Experimental group, normalized Log2(Readcount)					
	CE	RUN	H-RUN	LD	L-RUN	Log2FoldChange
Penk	10.5185718	11.3151772	11.2947862	10.4604868	11.3656483	0.834299407
Ogn	8.89979554	8.68861924	8.27249806	8.95020152	8.99652119	-0.677703452
Htra4	8.62509395	9.16810414	9.19542518	8.62401132	9.15061246	0.571413856
Igfbp5	12.6250156	12.1621899	12.1132248	12.6638244	12.1812405	-0.550599528
Sgk1	10.5992422	10.8809697	11.0816294	10.5356237	10.7945364	0.546005706
Grik3	10.4705266	9.9627263	9.88238656	10.4180034	10.0457097	-0.535616813
Dcn	9.82196785	9.33888101	8.93111531	9.45348581	9.73966479	-0.5223705
Fibcd1	10.659663	9.89081971	9.83200125	10.3510432	9.89522499	-0.519041991
Rprm	10.8410639	11.0629893	11.038455	10.5274124	11.0649474	0.511042639
Tmem181c-ps	11.8043991	11.685039	11.4228194	11.9220379	11.8106834	-0.499218517
Igfbp6	8.22917694	8.58009693	8.58451369	8.12605239	8.68367792	0.458461297
Pappa	7.14314488	7.49569082	7.61415581	7.15743364	7.27908444	0.45672217
Plekha2	11.7764956	12.2498475	12.2953989	11.8391995	12.1760193	0.456199366
Nos1	9.19258492	8.74407151	8.86874294	9.32254116	8.58458017	-0.453798216
Lgr6	9.05169574	8.9468912	8.99703545	8.54646686	8.87011006	0.450568596
Gan	7.28792719	7.14842509	7.36233145	6.91291598	6.79751515	0.44941547
Glt8d2	8.66268312	8.46432575	8.38234017	8.81881817	8.4252359	-0.436477995
Hrk	11.1289764	10.8266844	10.8371433	11.2723177	10.9245761	-0.435174456
Drd1a	8.17449717	8.56101026	8.63940785	8.20668292	8.44640589	0.432724935
Parm1	8.81094488	8.35539522	8.4446966	8.87501307	8.29996834	-0.430316476
Robo3	10.7194471	11.2040824	11.1716964	10.7447859	11.2695991	0.426910508
Tgfbr1	10.3891845	10.3337058	10.2381644	10.6624879	10.3904164	-0.424323542
Cdhr1	6.81358305	6.75146447	6.61525308	7.03642654	6.76695932	-0.421173456
Lars2	11.6651947	12.0404241	12.1987914	11.7783922	12.2622104	0.420399217
Nptx2	9.50701881	9.82123668	9.91664056	9.49684946	9.70008315	0.419791099
Fat4	11.6970101	11.3291672	11.3240808	11.7433672	11.4088816	-0.419286349
Rgs4	12.0114453	12.4003986	12.4463603	12.0299143	12.4060789	0.416445988
Kcnf1	10.31936	9.92108892	9.79511573	10.2082092	10.0003256	-0.413093437
Bok	10.0789539	10.4360326	10.6186267	10.2086215	10.2537167	0.410005165
Kitl	10.4541834	10.1989132	10.1867716	10.5946865	10.3170766	-0.407914949
Lrrc10b	10.6918985	11.0061206	11.120789	10.7153636	10.9334387	0.405425364
C1ql2	11.859996	12.061984	12.0990124	11.6948471	12.0651473	0.40416531
Kcnj16	8.94640401	8.58438949	8.5090532	8.91214992	8.77190327	-0.403096715
Gabra3	9.60584152	9.17536392	9.03308719	9.43571509	9.28797691	-0.402627901
Cd74	5.7228292	5.92896888	5.75243068	6.15474772	6.04607055	-0.402317048
Matn2	10.0883917	9.68608501	9.76553411	10.1663125	9.64121488	-0.400778374
Trnp1	11.3065425	11.4679815	11.5493963	11.1488296	11.4296282	0.400566672
Itpr1	12.4216037	12.3090912	12.3890718	12.7892696	12.184442	-0.400197874
Serinc2	7.79717218	8.1826361	8.19213146	7.79274925	8.15684879	0.399382216

Ptgs2	9.37381351	9.60451506	9.7233431	9.324603	9.42116365	0.398740098
2310003H01Rik	9.42522896	9.41600641	9.3429196	9.73785689	9.44904373	-0.394937289
Iglon5	10.3119159	10.497299	10.554441	10.1616942	10.5711904	0.392746828
Kcnq3	8.85727965	8.59921884	8.74723548	9.13933245	8.4509077	-0.392096969
Nuak1	10.1211215	10.0130633	9.9598556	10.3508027	9.99636516	-0.390947096
Vwa3a	9.76337316	9.50161614	9.46431759	9.85442321	9.5670256	-0.390105617
Kcnj2	9.90878035	10.2170165	10.3296064	9.94185899	10.099949	0.387747369
Ppl	8.67517519	9.00944355	8.96484192	8.57961031	9.02176922	0.385231614
Sh3bgrl3	11.7860774	11.9784827	11.9625349	11.5787142	12.0101768	0.383820743
Hlf	12.1387122	11.9322079	11.8928466	12.2736823	12.021205	-0.380835723
Ptpru	9.10229144	8.74544336	8.66294603	9.04142167	8.75074684	-0.378475646
E130008D07Rik	6.75865186	6.69379608	6.76002277	7.13675844	6.70471479	-0.376735667
Eif2c2	10.2864465	10.059634	10.1441487	10.5207837	9.90047943	-0.376635022
Ccbe1	9.85710057	9.59014459	9.66974775	10.0457353	9.54358949	-0.375987514
A730098P11Rik	9.68943119	9.52360254	9.34128094	9.71660349	9.80343346	-0.375322549
2900052N01Rik	9.66523454	9.60773691	9.45931938	9.83324772	9.650311	-0.373928337
Dsg2	8.66057339	8.64905645	8.51684197	8.88090315	8.7670567	-0.364061181
Mfsd4	11.017841	11.1010487	11.2513814	10.8883581	10.8226257	0.363023359
Bdnf	11.0798123	11.326044	11.4320423	11.0703317	11.263984	0.361710666
1500012F01Rik	9.29469969	9.6577612	9.63147572	9.27015275	9.62957111	0.361322969
Hspa5	13.2642082	13.2551312	13.0945491	13.4551812	13.3384413	-0.360632099
Blnk	8.08160159	8.30048418	8.36083199	8.00230269	8.15125795	0.358529299
Bhlhe40	10.9581102	10.8380831	10.7468253	11.1050407	10.8013987	-0.358215384
Darc	9.93472847	10.2210355	10.1775028	9.81943169	10.1096243	0.358071148
D430041D05Rik	12.6911234	12.4557608	12.468205	12.8253322	12.4560397	-0.357127224
Kif17	9.86316237	10.0886073	10.1089328	9.75243764	10.057612	0.356495165
Gpnmb	7.80010203	8.16922518	8.20062115	7.84556521	7.99460572	0.355055933
Elfn2	11.4172822	11.0100583	11.0653132	11.4196548	10.9146753	-0.354341676
Mid1	9.11291512	9.1448685	9.35758607	9.00355127	8.87394783	0.354034802
Atp2b4	12.285273	11.8573795	11.8452958	12.1978527	11.9624967	-0.352556954
Frrs1l	11.8747906	11.61003	11.7539126	12.1060602	11.4448769	-0.352147613
Dnajc3	10.6928311	10.6760295	10.6009843	10.9478827	10.6951276	-0.34689838
Synm	10.4025313	10.2567855	10.291512	10.6383304	10.1495119	-0.346818427
Nr1d2	11.5158144	11.3233077	11.3443423	11.6897983	11.3156418	-0.345455968
Fxyd7	8.90266603	9.07335297	9.17969013	8.83445935	8.92835962	0.345230774
Gstp1	11.3960183	11.3647691	11.4128975	11.0692446	11.3991357	0.343652886
Kif26b	9.08614888	8.93488454	9.00928071	9.35031291	8.80303563	-0.341032199
Hist1h1c	8.84932687	9.20013008	9.24387543	8.90439846	9.17529656	0.339476979
Zfp46	10.9083657	10.8935085	11.0157047	10.6789833	10.7064435	0.336721343
Daglb	10.1962157	10.2824386	10.4039247	10.0680764	10.2328714	0.335848283
Setbp1	10.4495926	10.2679822	10.3188744	10.6527648	10.2619389	-0.333890386
Ecm2	8.67250954	8.45252774	8.42778953	8.76139401	8.50246211	-0.333604481
Upp1	6.813165	6.61517116	6.54412948	6.87737185	6.64373507	-0.333242373

Zcchc5	7.52070523	7.26795345	7.22960954	7.56284892	7.32578586	-0.333239376
Grin3a	9.98888787	9.65533852	9.62400293	9.95659736	9.63887838	-0.33259443
Alg10b	9.31231778	9.23731369	9.30365096	9.63309167	9.071627	-0.329440718
Tmem181a	9.61679315	9.33658501	9.28245271	9.61090465	9.46227803	-0.32845194
Krt9	8.93861404	9.25436295	9.19510369	8.86777134	9.17241344	0.327332353
Amotl1	8.94349695	8.75727826	8.74987008	9.07540104	8.91352591	-0.325530964
Prmt8	10.7441116	10.7511655	10.7936674	11.1187103	10.6741466	-0.325042936
Nrgn	15.2276264	15.3376665	15.3905698	15.0676206	15.3681279	0.322949175
Frzb	10.3856732	10.2775485	10.2331735	10.5560642	10.3406985	-0.322890714
Megf9	10.1242169	9.91377859	10.1026799	10.4203484	9.66534183	-0.317668498
Etv1	10.7227528	10.3424413	10.3297669	10.6470704	10.3831355	-0.317303481
Rn45s	14.0768613	14.3771818	14.5809235	14.2639629	14.660353	0.316960593
Htr2c	9.1219739	8.50673857	8.33497652	8.65116853	8.76818293	-0.316192008
6030419C18Rik	12.4486048	12.4740934	12.5033042	12.1874247	12.4227914	0.315879504
Adamts1	9.27817213	9.11573617	9.16137056	9.47692312	9.12797064	-0.315552562
Tenm1	9.21663243	8.86597415	9.00688795	9.32212959	8.7629292	-0.315241634
Rhbdl3	9.06458449	8.69481657	8.60549411	8.9205717	8.8118643	-0.315077591
Acvr1c	10.2380195	10.5331088	10.6514955	10.3393285	10.4808638	0.31216698
Ypel4	10.1257298	10.3435142	10.3471169	10.0360238	10.4203434	0.311093032
Glp2r	7.1227972	7.24791055	7.24547342	6.93559919	7.12816219	0.309874231
Trpm3	10.8676342	10.6603474	10.8110026	11.1204239	10.4724765	-0.309421312
Tspan18	10.7220934	10.4447651	10.3677862	10.6759783	10.5184485	-0.308192147
1110008P14Rik	10.355522	10.4861515	10.5119266	10.2041234	10.4219171	0.307803264
Epha7	13.0960061	13.2333198	13.4270091	13.1197129	13.0317736	0.307296174
Rab40b	11.4771613	11.7271236	11.758537	11.451827	11.805146	0.306709955
Csdc2	11.6004372	11.7811167	11.7578108	11.453191	11.7941274	0.30461975
Gpr101	8.39154691	7.871618	7.56087349	7.86525083	8.10170773	-0.304377347
Adcyap1r1	12.6580862	12.4698067	12.4366222	12.7407524	12.463307	-0.304130209
Prr16	7.63896973	7.45487416	7.43336037	7.73470931	7.46756682	-0.301348939
Per3	11.2603313	11.1671695	11.1289076	11.4290461	11.1513047	-0.300138544
